# Poisson Variational Autoencoder

**Published:** 2024-12-09

**Authors:** Hadi Vafaii, Dekel Galor, Jacob L. Yates

**Affiliations:** 1 UC Berkeley

## Abstract

Variational autoencoders (VAEs) employ Bayesian inference to interpret sensory inputs, mirroring processes that occur in primate vision across both ventral [1] and dorsal [2] pathways. Despite their success, traditional VAEs rely on continuous latent variables, which deviates sharply from the discrete nature of biological neurons. Here, we developed the Poisson VAE (𝒫-VAE), a novel architecture that combines principles of predictive coding with a VAE that encodes inputs into discrete spike counts. Combining Poisson-distributed latent variables with predictive coding introduces a metabolic cost term in the model loss function, suggesting a relationship with sparse coding which we verify empirically. Additionally, we analyze the geometry of learned representations, contrasting the 𝒫-VAE to alternative VAE models. We find that the 𝒫-VAE encodes its inputs in relatively higher dimensions, facilitating linear separability of categories in a downstream classification task with a much better (5×) sample efficiency. Our work provides an interpretable computational framework to study brain-like sensory processing and paves the way for a deeper understanding of perception as an inferential process.

## 1 Introduction

The study of artificial neural networks (ANN) and neuroscience has always been closely linked, driving advancements in both fields [5–10]. Despite the close proximity of the two fields, most ANN models deviate substantially from biological brains [11, 12]. A major challenge is designing models that not only perform well computationally but also exhibit “brain-like” structure and function. This is seen both as a goal for improving ANNs [13–15], and better understanding biological brains [8, 9, 16–19], which has recently been referred to as the *neuroconnectionist* research programme [20].

Drawing from neuroscience, a major guiding idea is that perception is a process of inference [21, 22], where the brain constructs a representation of the external world by inferring the causes of sensory inputs [23–26]. This concept is mirrored in “generative AI” where models learn the generative process underlying their inputs [27–29]. However, in this vein, there is a tension between small well-understood models that are directly inspired by cortex, such as sparse coding [3] and predictive coding [30], and deep generative models that perform well [31–34].

The variational autoencoder (VAE; [35, 36]) model family is a promising candidate for neuroconnectionist goals for multiple reasons. First, VAEs learn probabilistic generative models of their inputs and are grounded in Bayesian probability theory, providing a solid theoretical foundation that directly incorporates the concept of perceptual inference [10, 22]. Second, the VAE model family, specifically hierarchical VAEs, is broad with other generative models, such as diffusion models, understood as special cases of hierarchical VAEs [37–39]. Finally, VAEs learn representations that are similar to cortex [1, 2, 40], exhibit cortex-like topographic organization [41, 42], and make perceptual errors that mimic those of humans [43], indicating a significant degree of neural, organizational, and psychophysical alignment with the brain.

However, standard VAEs diverge from brains in the way they encode information. Biological neurons fire all-or-none action potentials [44], and are thought to represent information via firing rate [45–49]. These firing rates must be positive and generate discrete “spike” counts, which exhibit conditionally Poisson-like statistics in small counting windows [49–51]. In contrast, VAEs are typically parameterized with real-valued, continuous, Gaussian distributions [52].

### Contributions.

**Figure 1:**
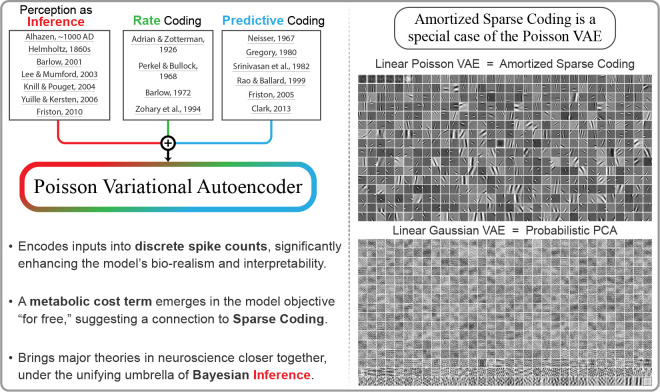
Graphical abstract. Introducing the Poisson Variational Autoencoder (𝒫-VAE), which draws on key concepts in neuroscience. When trained on natural image patches, 𝒫-VAE with a linear decoder develops Gabor-like feature selectivity, reminiscent of Sparse Coding [3]. In sharp contrast, the standard Gaussian VAE learns the principal components [4]. Our code, data, and model checkpoints are available at this repository: https://github.com/hadivafaii/PoissonVAE

**Figure 2:**
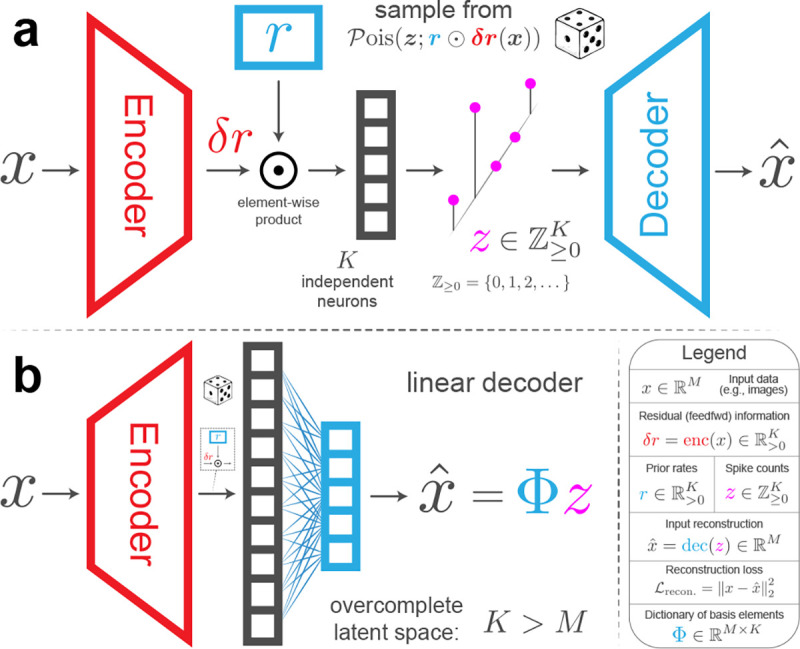
(**a**) Model architecture. Colored shapes indicate learnable model parameters, including the prior firing rates, r. We color code the model’s inference and generative components using red and blue, respectively. The 𝒫-VAE encodes its inputs in discrete spike counts, z, significantly enhancing its biological realism. (**b**) “Amortized Sparse Coding” is a special case within the 𝒫-VAE model family: it’s a 𝒫-VAE with a linear decoder and an overcomplete latent space.

In this work, we address this discrepancy by introducing the Poisson Variational Autoencoder (𝒫-VAE), a novel architecture that combines perceptual inference with two other inspirations from neuroscience (Fig. 1). First, that information is encoded in the rates of discrete spike counts, which are approximately Poisson-distributed on short time intervals. And second, that feedforward connections encode deviations from expectations contained in feedback connections (Fig. 2a; [30, 53]). We introduce a reparameterization trick for Poisson samples (Algorithm 1), and derive the evidence lower bound (ELBO) objective for the 𝒫-VAE (eq. (3)). Overall, we believe 𝒫-VAE introduces a promising new model at the intersection of computational neuroscience and machine learning that offers several appealing features over existing VAE architectures:

- The 𝒫-VAE loss derivation (eq. (3)) naturally results in a metabolic cost term that penalizes high firing rates, such that 𝒫-VAE with a linear decoder implements amortized sparse coding (Fig. 2b). We validate this prediction empirically.
- 𝒫-VAE largely avoids the prevalent posterior collapse issue, maintaining many more active latents compared to alternative VAE models (Table 1), especially the continuous ones.
- 𝒫-VAE encodes its inputs in relatively higher dimensions, facilitating linear separability of categories in a downstream classification task with a much better (5×) sample efficiency.

We evaluate these results on two natural image datasets and MNIST. The 𝒫-VAE paves the way for the future development of interpretable hierarchical models that perform “brain-like” inference.

## 2 Background & Related work

### Perception as inference: connections to neuroscience and machine learning.

A centuries-old idea [21, 22], “perception as inference” argues that coherent perception of the world results from the unconscious inference over the causes of the senses. In other words, the brain learns a generative model of the sensory inputs. This has led to fruitful theoretical work in neuroscience [23, 54–56] and machine learning [57, 58], including VAEs [52]. See Marino [10] for a review.

### Efficient, predictive, and sparse coding.

Another longstanding idea in neuroscience is that brains are adapted to the statistics of the environment. Efficient coding states that brains represent as much information about the environment as possible while minimizing neural resource use [59, 60].

Predictive coding [30, 61, 62] postulates that the brain generates a statistical prediction of its inputs, with feedforward networks carrying only the prediction errors or unexplained information [63]. More recently, ANNs based on predictive coding have been shown to capture a wide range of phenomena in biological neurons across the visual system [64, 65]. More broadly, prediction in time has emerged as an objective that lends itself to brain-like representations [66, 67].

Sparse coding (SC) is directly inspired by efficient coding, aiming to explain inputs as sparsely as possible [47, 68]. SC was the first unsupervised model to learn representations closely resembling the receptive fields of V1 neurons [3] and predicts an array of empirical features of neural activity [69–79]. SC is formalized with a generative model where neural activations z are sampled from a sparsity-inducing prior, z~p(z), and the input image x is reconstructed as a linear combination of basis vectors Φ, plus additive Gaussian noise, $\overset{ˆ}{x}=\Phi z+\varepsilon$. The SC loss is as follows:

(1) $${ℒ}_{\text{SparseCoding}}(x;\Phi ,z)=\|x-\Phi z{\|}_{2}^{2}+\beta \|z{\|}_{1}.$$

Commonly used algorithms for sparse coding include the locally competitive algorithm (LCA; [80]), which is a biologically plausible algorithm to optimize eq. (1), and iterative shrinkage-thresholding algorithm (ISTA; [81, 82]), which has shown robust performance in learning sparse codes given a fixed dictionary Φ.

### VAE objective.

VAEs define a probabilistic generative model p(x,z), where x denotes the observed data and z are some latent variables. The generative process samples z from a prior distribution p(z) and then generates the observed data x from the conditional distribution $p_{\theta }(x|z)$, also known as the “decoder”. The “encoder”, $q_{\phi }(z|x)$, performs approximate inference on the inputs. Model parameters are learned by maximizing the evidence lower bound (ELBO) objective, which is derived from variational inference (see appendix B for the full set of derivations). The ELBO is given by:

(2) $$log\;p(x)\geq E_{q_{\phi }(z|x)}\left[log\;p_{\theta }(x|z)\right]-{𝒟}_{KL}\left(q_{\phi }(z|x)\|p(z)\right)={ℒ}_{VAE}(x;\theta ,\phi ).$$

The first term captures the reconstruction performance of the decoder, and the second term, the “KL term,” captures the divergence of the approximate posterior from the prior.

The specific form of these distributions is up to the practitioner. In standard VAEs, factorized Gaussians are typically used: $q=𝒩\left(z;\mu (x),\sigma^{2}(x)\right)$ and p=𝒩(z;0,1). The likelihood, $p_{\theta }(x|z)$, is also typically modeled as a Gaussian conditioned on a parameterized neural network ${dec}_{\theta }(z)$.

### Amortized inference in VAEs.

A major contribution of VAEs is the idea of amortizing inference over the latents z with a black box ANN [83, 84]. “Amortized” inference borrows a term from finance to capture the idea of spreading out costs—here, the cost of performing inference over multiple samples. In amortized inference, a neural network learns (during training) how to map a data sample to a distribution over latent variables given the sample. The cost is paid during training, but the trained model can then be used to perform inference on future samples efficiently. It has been argued that the brain performs amortized inference for computational efficiency [85].

### VAEs connection to biology.

VAEs have been shown to contain individual latents that resemble neurons, capturing a wide range of the phenomena observed in visual cortical areas [40] and human perceptual judgments [43]. Like many other ANN models [86, 87], VAEs have been found to learn representations that are predictive of single-neuron activity in both the ventral [1] and dorsal [2] streams. However, unlike most ANNs, the mapping from certain VAEs to neural activity is incredibly sparse, even one-to-one in some cases [1, 2].

### Discrete VAEs.

VAEs with discrete latent spaces, such as VQ-VAE [88] and Categorical VAE [89], are designed to capture complex data structures by mapping inputs to a finite set of latent variables. Unlike traditional VAEs that use continuous latent spaces, these models leverage discrete representations to enhance interpretability and can yield high performance with lower capacity [90].

**Algorithm 1:** Reparameterized sampling (rsample) for Poisson distribution.

	**Input:**		
	$\lambda \in {\mathbb{R}}_{>0}^{B\times K}$	# rate parameter; *B*, batch size; *K*, latent dimensionality	
	*n*_exp	# number of exponential samples to generate	
	temperature	# controls the sharpness of the thresholding	
1:	**procedure** Rsample(**λ**, *n*_exp, temperature)		
2:	Exp ← Exponential(**λ**)		⊳ create exponential distribution
3:	Δt ← Exp.rsample((*n*_exp,))		⊳ sample inter-event times, Δ*t* : [*n*_exp × *B* × *K*]
4:	times ← cumsum(Δ*t*, dim=0)		⊳ compute arrival times, same shape as Δ*t*
5:	$\text{indicator}\;\leftarrow \text{sigmoid}\left(\frac{1-\text{times}}{\text{temperature}}\right)$		⊳ soft indicator for events within unit time
6:	*z* ← sum(indicator, dim=0)		⊳ event counts, or number of spikes, *z* : [*B* × *K*]
7:	**return** *z*		
8:	**end procedure**		

### VAEs connection to sparse coding.

Previous work has attempted to connect sparse coding and VAEs directly [91–93], with each approaching the problem differently. Geadah et al. [91] introduced sparsity-inducing priors (such as Laplace or Cauchy) and a linear decoder with an overcomplete latent space. Tonolini et al. [92] introduced a spike and slab prior into a modified ELBO, and Xiao et al. [93] added a sparse coding layer learned by ISTA to the latent space of a VQ-VAE. Notably, none of the three ended up minimizing the sparse coding loss. Two of the three maintain the linear generative model with an overcomplete latent space, but the ELBO in both requires an additional approximation step for the KL term [91, 92].

## 3 Introducing the Poisson Variational Autoencoder (𝒫-VAE)

Our main contribution is integrating Poisson-distributed latents into VAEs, where both the approximate posterior and the prior are parameterized as Poisson distributions. Critically, the latents z are no longer continuous variables, but rather they are discrete spike counts. To perform inference over discrete latents, we introduce a Poisson reparameterization trick. We then derive the KL term and obtain the full 𝒫-VAE objective.

### Poisson reparameterization trick.

For a homogeneous Poisson process [94–96], given a window size ∆t=1, and rate λ, we can generate Poisson distributed counts by drawing randomly distributed wait-times from an exponential distribution with mean 1/λ and counting all events where the cumulative time is less than 1. Because the exponential distribution is trivially reparameterized [35], and PyTorch contains an implementation [97], we need only to approximate the hard threshold for comparing cumulative wait times with the window size. We accomplish this by replacing the indicator function with a sigmoid as in refs. [89, 98].

Algorithm 1 demonstrates the steps: Given a matrix of rates λ, sample n_exp wait times $t_{1},t_{2},\ldots t_{n\_exp}$ for each element of λ by sampling from an exponential distribution with mean 1/λ. We then calculate the cumulative event times $S\left(n_{-}exp\right)=\sum_{j=1}^{n_{-}exp}\;t_{j}$, pass them through a sigmoid $\sigma \left(\frac{1-S}{\text{temperature}}\right)$, and sum over samples to get event counts, z. The temperature controls the sharpness of the thresholding. We adaptively scale the number of samples, n_exp, by keeping track of the maximum rate in each batch, $\lambda_{max}$, and then use the inverse cumulative density function (cdf) for Poisson to find the number of samples, n_exp, such that $cdf\left(n_{-}exp;\lambda_{max}\right)=0.99999$.

**Figure 3:**
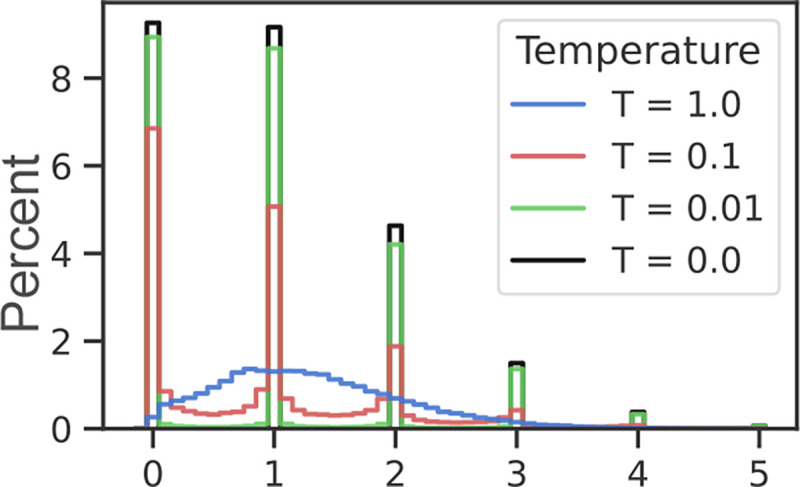
Relaxed Poisson distribution. Samples are drawn using Algorithm 1 for λ=1. At non-zero temperatures, samples are non-integer, but approach the true Poisson distribution as T→0.

At non-zero temperatures, our parameterization algorithm provides a continuous relaxation of the Poisson distribution. Figure 3 shows histograms of samples drawn using Algorithm 1 for rate λ=1 and temperatures T=1.0,0.1,0.01, and 0. The latter case (T=0, true Poisson) is equivalent to torch.poisson().

### 𝒫-VAE architecture and residual parameterization.

The architecture of 𝒫-VAE captures the interactions between feedforward and feedback connections that are present in all visual cortical areas [99, 100]. Feedforward areas carry sensory information and feedback connections are thought to carry modulatory signals such as attention [53] or prediction [30], which interact multiplicatively with feedforward inputs [53, 101].

𝒫-VAE embodies this idea by having the posterior rates depend on the prior, such that $r_{\text{prior}}=r$ and $r_{\text{post.}}=r⊙\delta r(x)$, where ⊙ is the Hadamard (element-wise) product. The prior rates, $r\in R^{K}$, are learnable parameters that capture expectations about the statistics of the input. The encoder outputs, $\delta r(x)\in R^{K}$, capture *deviations* from the prior. Thus, 𝒫-VAE models the interaction between prior expectations, and deviations from them, in a multiplicative and symmetric way. This results in a posterior, q(z∣x)=𝒫ois(z;r⊙δr(x)), and prior, p(z)=𝒫ois(z;r), where z is the spike count variable and $𝒫ois(z;\lambda )=\lambda^{z}e^{-\lambda }/z!$ is the Poisson distribution. Notably, this multiplicative relationship is maximally general, as any pair of positive variables, $r_{\text{prior}}$, and $r_{\text{post.}}$ can be expressed as a base variable, $r:=r_{\text{prior}}$, multiplied by their relative ratio, $\delta r:=r_{\text{post.}}/r$. See Fig. 2a.

### 𝒫-VAE loss function.

For a comprehensive derivation of the 𝒫-VAE objective, see appendix B. Here, we report the final result:

(3) $${ℒ}_{PVAE}=E_{z\sim 𝒫ois(z;r⊙\delta r)}\left[\|x-dec(z){\|}_{2}^{2}\right]+\sum_{i=1}^{K}r_{i}f\left(\delta r_{i}\right),$$

where dec(⋅) is the decoder neural network, and $f\left(y\right)\;:=1-y+y\;log\;y$ (see supplementary Fig. 6).

### 𝒫-VAE relationship to sparse coding.

The KL term in eq. (3) penalizes firing rates. Both r and δr are positive by definition, and f(y)≥0, strongly resembling the sparsity penalty in Olshausen and Field [3]. To make this connection more explicit, we make two additional assumptions (Fig.2b):

1. The decoder is a linear generative model: $\overset{ˆ}{x}=\Phi z$, with $x\in R^{M}$ and $\Phi \in R^{M\times K}$.
2. The latent space is overcomplete: K>M.

Because both $E_{z\sim 𝒫ois(z;\lambda )}\left[z_{i}\right]$ and $E_{z\sim 𝒫ois(z;\lambda )}\left[z_{i}z_{j}\right]$ have closed-form solutions (eq. (22)), the reconstruction term in eq. (3) can be computed analytically for a linear decoder, resulting in:

(4) $${ℒ}_{\text{SC-PVAE}}(x;\delta r,r,\Phi )=\|x-\Phi \lambda {\|}_{2}^{2}+\lambda^{T}diag\left(\Phi^{T}\Phi \right)+\beta \sum_{i=1}^{K}r_{i}f\left(\delta r_{i}\right).$$

where λ=r⊙δr(x) are the posterior firing rates, f(y) is defined as above, and β is a hyperparameter that scales the contribution of the KL term [102], and changes the sparsity penalty for the 𝒫-VAE.

The relationship between the linear 𝒫-VAE loss (eq. (4)) and the sparse coding loss (eq. (1)) can now be seen. Both contain a term that minimizes the squared error of the reconstruction and a term (two terms for 𝒫-VAE) that penalizes non-zero firing rates. Unlike prior work that directly implemented amortized sparse coding [91, 92], here the activity penalty naturally emerges from the derivations, and the only additional assumption was an overcomplete linear generative model. The inference is accomplished using a parameterized feed-forward neural network, δr(x), thus, it is amortized [83]. We call this specific case of 𝒫-VAE “Amortized Sparse Coding” (Fig. 2b).

Note that a closed-form derivation of the reconstruction term is possible for any VAE with a linear decoder and a generating distribution that has a mean and variance (see eq. (21)).

This closed-form expression of the loss given a linear decoder is useful because we can see how different parameters contribute to the loss. Furthermore, we can compute gradients of the whole loss exactly, and use this to evaluate our Poisson reparameterization.

## 4 Experiments

To evaluate the 𝒫-VAE, we perform three sets of experiments. First, we utilize the theoretical results for a linear decoder (eqs. (4) and (21)) to test the effectiveness of our reparameterization algorithm. We compare to alternative VAE models with established reparameterization tricks (e.g., Gaussian).

Second, to confirm 𝒫-VAE with a linear decoder not only resembles amortized sparse coding but practically performs like sparse coding, we compare to standard and well-established sparse coding algorithms such as the locally competitive algorithm (LCA; [80]) and the widely-used iterative shrinkage-thresholding algorithm (ISTA; [81, 82]) to see if 𝒫-VAE reproduces their results.

Third, we test the 𝒫-VAE in a generic representation learning context and evaluate the geometry of learned representations for downstream tasks. For these experiments, both the encoder and decoder’s architecture is a ResNet (see appendix C for full architecture and training details).

### Architecture notation.

We experimented with both convolutional and linear architectures. We highlight the encoder and decoder networks using red and blue, respectively. We use the ⟨enc|dec⟩ convention to clearly specify which architecture type was used. For example, ⟨conv|lin⟩ represents a model with a convolutional encoder and a linear decoder. Using this notation, we note that ⟨lin|lin⟩ and ⟨conv|lin⟩ architectures were used for the first and second sets of experiments, while ⟨conv|conv⟩ architectures were employed for the third.

### Alternative models.

We compare 𝒫-VAE to both discrete and continuous VAEs (Table 1). Other than the traditional Gaussian, we compare to Laplace-distributed VAEs because previous work found the Laplace distribution supported robust sparse representations [40, 91]. Additionally, we compare to Categorical VAEs, trained using the Gumbel-Softmax trick [89, 98]. We use PyTorch’s implementation which is based on Maddison et al. [98].

Finally, we test models where Gaussian latents are passed through an activation function before passing to the decoder. We call these models $𝒢-{VAE}_{+act}$, where act∈{relu,exp}, capturing other families of distributions (truncated Gaussian and log-normal). We include these to test the hypothesis that positive constraints (and not discrete latents) are the key contribution of Poisson [103].

### Datasets.

For sparse coding results, we use 101 natural images from the van Hateren dataset [104]. We tile the images to extract 16 × 16 patches and apply whitening and contrast normalization, as is typically done in sparse coding literature [3, 105]. To test the generalizability of our sparse coding results, we repeat these steps on CIFAR10 [106], a dataset we call CIFAR_16×16_. For the general representation learning results, we use MNIST. See appendix C for additional details.

### Statistical tests.

In the VAE literature, it is known that random seeds can have a large effect compared to architecture or regularization [108]. Therefore, we train each configuration using 5 different random initializations. We report 99% confidence intervals throughout, and perform paired *t*-tests, reporting significance for p<0.01 (FDR corrected using the Benjamini-Hochberg method).

### Evaluating the Poisson reparameterization algorithm.

𝒫-VAE with a linear decoder has a closed form solution (eq. (4)), which lets us evaluate how well our reparameterized gradients perform compared to the exact ones. We compare our results to the gold-standard Gaussian (Table 2), as well as Categorical and Laplace VAEs (supplementary Table 5). In Table 2, we report the percent performance drop relative to the best fit, enabling meaningful comparisons across architectures and datasets. Monte Carlo sampling with Poisson reparameterization closely matches exact inference just like established methods for Gaussian and Laplace. In contrast, the straight-through (ST; [107]) estimator performs poorly (Table 2; see also supplementary Fig. 7).

### Annealing the temperature.

The temperature parameter (T) is a crucial hyperparameter in our Poisson reparameterization trick (Algorithm 1). To assess its impact, we followed standard practice [89] and annealed T during the first half of training, starting from a large value ($T_{\text{start}}=1$) and gradually decreasing it to a small value ($T_{\text{final}}=0.05$ in the main paper). Figure 9 shows the performance on the van Hateren dataset as a function of various $T_{\text{final}}$, two architectures (⟨lin|lin⟩ and ⟨conv|lin⟩), as well as two annealing schedules (linear vs. exponential; see inset). We find that final temperatures $T_{\text{final}}\leq 0.1$ and either annealing strategy work well.

During training, we maintain T>0, which results in continuous (floating) latent variables, z. At test time, we set T=0 to produce genuine integer Poisson samples. Crucially, all reported results use T=0 at test time. We also explored a “hard-forward” scheme during the latter half of training, where T remains nonzero only in the backward pass. This *surrogate gradients* approach provides integer latents in the forward pass but, somewhat unexpectedly, underperformed our “relaxed Poisson” method (Fig. 9). These findings suggest that surrogate gradient methods might benefit from relaxing the hard-forward strategy during training. We believe this observation will be of particular interest to the spiking neural network community, which often relies on surrogate gradients for training.

### The 𝒫-VAE learns basis vectors similar to those from sparse coding.

**Figure 4:**
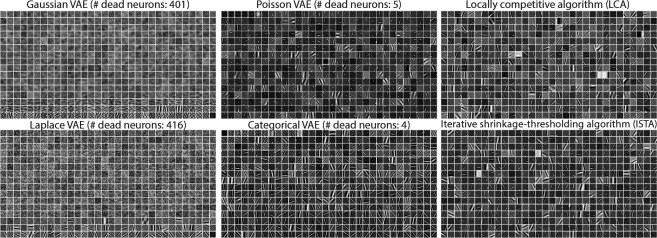
Learned basis elements for various ⟨lin|lin⟩ VAEs (first two columns) and standard sparse coding models (last column). There are a total of K=512 elements, each made of 16 ×16 = 256 pixels (i.e., $\Phi \in R^{256\times 512}$). Features are ordered from top-left to bottom-right, in ascending order of their associated KL divergence (𝒫-VAE, 𝒢-VAE, ℒ-VAE), or the magnitude of posterior logits (𝒞-VAE). The sparse coding results (LCA and ISTA) are ordered randomly.

A major result from sparse coding is that it learns basis vectors (dictionaries) that resemble the “Gabor-like” receptive fields of cortical neurons [3, 109, 110]. Inspecting the dictionaries learned by different models demonstrates this is not trivial (Fig. 4). As expected from theoretical results [4], 𝒢-VAE (top left) learn probabilistic PCA, but with many noisy elements. As demonstrated previously [40, 91], ℒ-VAE (lower left) learn Gabor-like elements. However, there are a large number of noisy basis vectors. It is of note that previous work did not show complete dictionaries for their results with Laplace latents [40, 91]. In contrast, 𝒫-VAE (top middle) learns Gabor-like filters that cover space, orientation, and spatial frequency. The quality is comparable to sparse coding dictionaries learned with LCA/ISTA (top/lower right panels). 𝒞-VAE also learns Gabors, although there are significantly more noisy basis elements.

### The 𝒫-VAE avoids posterior collapse.

A striking feature of Fig. 4 is the sheer number of noisy basis vectors for both continuous VAEs (𝒢-VAE, ℒ-VAE). We suspected this reflected dead neurons with vanishing KL, which is indicative of a collapsed latent dimension that’s no longer encoding information. To quantify this, we binned the distribution of KL values and thresholded the resulting distribution at discontinuous points (see supplemental Fig. 10). Table 3 shows the results of this analysis for all VAEs with valid KL terms. Across all datasets, both continuous VAEs suffered from large numbers of dead neurons, whereas 𝒫-VAE largely avoided this problem. On both natural image datasets, 𝒫-VAE had ~2% dead neurons compared to ~80% for 𝒢-VAE and ℒ-VAE. Having a more expressive encoder slightly increases this percentage, but a dramatic difference between 𝒫-VAE and continuous VAEs (𝒢-VAE, ℒ-VAE) persists.

### The 𝒫-VAE learns sparse representations.

To quantify whether 𝒫-VAE learns sparse representations, we compared our VAE models to sparse coding trained with LCA and ISTA and quantified the lifetime sparsity [69]. The lifetime sparsity of the *j*-th latent is:

(5) $$s_{j}={\left(1-\frac{1}{N}\right)}^{-1}\left(1-\frac{1}{N}\frac{{\left(\sum_{i}\;z_{ij}\right)}^{2}}{\sum_{i}\;z_{ij}^{2}}\right),$$

where N is the number of images, and $z_{ij}$ is sampled from the posterior for the *i*-th image. Intuitively, $s_{j}=1$ whenever neuron j responds to a single stimulus out of the entire set (highly selective). In contrast, $s_{j}=0$ whenever the neuron responds equally well to all stimuli indiscriminately.

**Figure 5:**
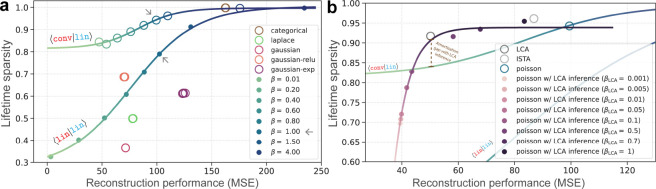
Reconstruction performance vs. sparsity of representations. (**a**) Results for the VAE model family. The curves are sigmoid fit to ⟨lin|lin⟩ and ⟨conv|lin⟩ 𝒫-VAE results across varying β values (β from eq. (4)). Empty circles correspond to ⟨conv|lin⟩ architectures. (**b**) Amortization gap for 𝒫-VAE (blue open circle) compared to sparse coding (LCA/ISTA). Solid points show results from applying the LCA inference algorithm to 𝒫-VAE basis vectors at different sparsity levels ($\beta_{LCA}$ from eq. (1)). The purple curve is a sigmoid fit, and curves from part (a) are also included for comparison.

Fig. 5a shows the reconstruction performance (MSE) compared to lifetime sparsity (s, eq. (5)) for all VAEs. Empty and solid circles represent ⟨conv|lin⟩ and ⟨lin|lin⟩ architectures, respectively. The 𝒢-VAE finds good reconstructions (MSE = 71*.*49) but with low sparsity (s=0.37). Because the 𝒫-VAE KL term explicitly penalizes rate (eq. (3)), we explored different β values for 𝒫-VAE with both ⟨lin|lin⟩ and ⟨conv|lin⟩ architectures (Fig. 5a, blue curves). This maps out rate-distortion curves, enabling us to compare the sparsity levels at which 𝒫-VAE matches 𝒢-VAE performance.

With a simpler (linear) encoder, ⟨lin|lin⟩ 𝒫-VAE matches ⟨conv|lin⟩ 𝒢-VAE performance while achieving 1*.*7× greater sparsity at β=0.6. A ⟨conv|lin⟩ 𝒫-VAE further increases this gap to 2.4× greater sparsity. Adding a relu activation to 𝒢-VAE also increases sparsity (s=0.69). By comparing ⟨lin|lin⟩ and ⟨conv|lin⟩ 𝒫-VAE models, we observe that enhancing encoder complexity for the same β=1 (gray arrows) preserves MSE performance while achieving greater sparsity. This highlights how amortization quality can significantly influence rate-distortion curves [33, 111–113].

Does 𝒫-VAE match the performance of traditional sparse coding trained with LCA or ISTA? Figure 5b compares 𝒫-VAE to sparse coding models that were trained using a wide range of hyperparameters, and the best models were selected for each class (appendix C). 𝒫-VAE achieves a similar sparsity to LCA and ISTA (s=0.94, 0*.*91, and 0*.*96, respectively), but the best LCA model drastically outperforms 𝒫-VAE on MSE for similar levels of sparsity. This suggests our convolutional encoder is struggling to close the amortization gap. To test this hypothesis, we performed LCA inference on basis elements learned by 𝒫-VAE (Fig. 5b curve/solid points). We explored a range of hyperparameters to determine whether the MSE improved for similar sparsity levels. Indeed, LCA inference using 𝒫-VAE dictionary was able to nearly match the performance of sparse coding LCA for similar levels of sparsity. This confirms our hypothesis that a large amortization gap remains for the specific encoder architectures we tested, highlighting the need for improved inference algorithms/architectures [112].

### The 𝒫-VAE is more sample efficient in downstream tasks.

To assess downstream performance, we trained ⟨conv|conv⟩ VAE models with a K=10 latent dimension on MNIST (see supplementary Fig. 12 for generated samples and reconstructions from these models). We then extracted representations from the trained encoders and evaluated their ability to classify MNIST digits. We define representations as mean vectors μ for continuous VAEs (𝒢-VAE, ℒ-VAE) following conventions in the VAE literature [108], and use log δr for 𝒫-VAE, and logits for 𝒞-VAE.

We split the MNIST validation set into two 5,000 sample sets, used as train/test sets for this task. We train K-nearest neighbors (KNN) classifiers with a varying number of limited supervised samples (N=200,1000,5000) drawn without replacement from the first set (train), to measure classification accuracy on the withheld set (test). KNN is nonparametric, and its performance is directly influenced by the geometry of representations by explicitly capturing the distance between encoded samples [114]. We find that using only N=200 samples, 𝒫-VAE achieves ~ 82% accuracy in held out data; whereas, 𝒢-VAE achieves the same level of accuracy at N=1000 samples (Table 4). By this measure, 𝒫-VAE is 5× more sample efficient. But from Alleman et al. [115], we know that the choice of activation function changes the geometry of learned representations. Therefore, we also tested 𝒢-VAE models with an activation function (relu and exp) applied to latents after sampling from the posterior. This biological constraint improved 𝒢-VAE, but it still underperformed 𝒫-VAE (Table 4). We also found this result held for higher dimensional latent spaces (supplementary Table 6).

In supplementary analyses (Fig. 11), we evaluated the representations using logistic regression trained on the full dataset. For larger latent dimensionalities (K=50,100), 𝒫-VAE outperformed all other VAEs, but at lower dimensionalities (K=10), it underperforms both 𝒢-VAE and ℒ-VAE.

### The 𝒫-VAE learns representations with higher dimensional geometry.

The preceding results are indicative of substantial differences in the geometry of the representations learned by 𝒫-VAE compared to other VAE families (Table 4). To test this more explicitly, we calculated the “shattering dimensionality” of the latent space [116–118]. Shattering dim measures the average accuracy over all possible pairwise classification tasks. This is called “shattering” because if the model shatters data points around into a high dimensional space, they will become more linearly separable. For MNIST with 10 classes, there are 105=252 possible classifications. We trained logistic regression on the entire training set to classify each of the 252 arbitrary splits and measured the average performance on the entire validation set. The far right column of Table 4 shows the measured shattering dims. For K=10, the shattering dim was significantly higher for discrete VAEs (𝒫-VAE, 𝒞-VAE). For higher dimensional latent spaces 𝒫-VAE strongly outperformed alternative models (Table 6).

## 5 Conclusions

In this paper, we introduced the 𝒫-VAE, a generative model that encodes inputs into discrete spike counts and unifies established theoretical concepts in neuroscience with modern machine learning. We introduced a Poisson reparameterization algorithm and derived the ELBO for Poisson-distributed latent variables. The 𝒫-VAE objective results in a KL term that penalizes firing rates, like sparse coding. We showed that 𝒫-VAE with a linear decoder reduces to amortized sparse coding. We evaluated the representations on downstream classification tasks and found that 𝒫-VAE encodes its inputs in a higher dimensional space, enabling good linear separability between classes.

### Limitations.

𝒫-VAE samples Poisson latents. Although this is inspired by the statistics of spike counts in the brain over short time intervals [50], there are deviations from Poisson throughout the cortex over longer time windows [51]. We discuss this point in appendix A. A second limitation is the amortization gap between our current implementation of 𝒫-VAE and traditional sparse coding. This could likely be closed with more expressive encoders [119] or through iterative inference [113, 120], but it is an open area of research [112].

### Neuroscience implications and future directions.

Like biological neurons, the P-VAE generates spikes. This non-negative, discrete representational form closely parallels neuronal spiking activity. Therefore, the 𝒫-VAE can be more directly compared to neuronal circuits than unconstrained, continuous VAEs. This analogy facilitates in silico perturbation experiments (e.g., “stimulating” or “silencing” P-VAE neurons) to mirror in vivo causal manipulations. It also allows applying methods like *Most Exciting Inputs* (MEI; [121]), which assume non-negative activations. Future work could explore hierarchical P-VAEs, finding a sweet spot between interpretability and performance. Overall, the biologically inspired representational form of P-VAE brings computational modeling closer to experimental neuroscience and opens new avenues for advancing NeuroAI research [13, 20].

## Supplementary Material

Supplement 1

## Figures and Tables

**Table 1:** Models considered in this paper.

Discrete		Continuous	
Poisson VAE (𝒫-VAE)	Categorical VAE (𝒞-VAE; [89, 98])	Gaussian VAE (𝒢-VAE; [35, 36])	Laplace VAE (ℒ-VAE; [40, 91])

**Table 2:** Reparameterized gradient estimators perform comparably to exact ones across datasets and encoder architectures (linear vs. convolutional). Exact gradients are only computable for linear decoders (see eqs. (21), (23) and (24)). Values represent percent drop in validation loss (lower is better), shown as mean±99% confidence interval calculated from *n* = 5 random initializations. The best-performing case was selected as the single best random seed for models of the same architecture and dataset across gradient methods (1 out of: 15 for 𝒫-VAE, 10 for 𝒢-VAE). See supplementary Fig. 7 for a visualization of the same data presented in this table. For actual loss values, see supplementary Table 5. EX: exact; MC: Monte Carlo; ST: straight-through [107].

Model		van Hateren		CIFAR_16×16_		MNIST	
		⟨lin|lin⟩	⟨conv|lin⟩	⟨lin|lin⟩	⟨conv|lin⟩	⟨lin|lin⟩	⟨conv|lin⟩

𝒫-VAE	EX	0.6±.5	0.1±.1	0.0±.1	0.0±.0	0.1±.1	0.5±.6
	MC	0.0±.1	0.7±.1	0.2±.0	0.5±.1	0.7±.4	0.9±.5
	ST	7.3±.1	10.5±.1	9.1±.1	12.5±.1	8.1±.3	11.8±.2

𝒢-VAE	EX	0.1±.1	0.0±.0	0.0±.1	0.0±.0	0.1±.2	0.1±.2
	MC	0.1±.1	0.0±.0	0.1±.1	0.0±.0	0.4±.1	0.3±.1

**Table 3:** Proportion of active neurons. All models considered in this table had a latent dimensionality of *K* = 512, with either ⟨lin|lin⟩ or ⟨conv|lin⟩ architectures. See also supplementary Fig. 10.

Model	van Hateren		CIFAR_16×16_		MNIST	
	linear	conv	linear	conv	linear	conv

𝒫-VAE	**0.984**±.011	**0.819**±.041	**0.999**±.002	**0.928**±.045	**0.537**±.008	**0.426**±.011
ℒ-VAE	0.188±.000	0.222±.003	0.193±.003	0.230±.000	0.027±.000	0.034±.002
𝒢-VAE	0.218±.003	0.246±.000	0.105±.008	0.246±.000	0.027±.000	0.031±.000

**Table 4:** Geometry of representations (*K* = 10 only; see Table 6 for the full set of results).

Latent dim.	Model	KNN classification (*N*, # labeled samples)			Shattering dim.
		*N* = 200	*N* = 1,000	*N* = 5,000	

*K* = 10	𝒫-VAE	**0.815**±.002	**0.919**±.001	**0.946**±.017	**0.797**±.009
	𝒞-VAE	0.705±.002	0.800±.002	0.853±.040	**0.795**±.006
	ℒ-VAE	0.757±.003	0.869±.002	**0.924**±.028	0.751±.008
	𝒢-VAE	0.673±.003	0.813±.002	0.891±.033	0.758±.007
	𝒢-VAE _+relu_	0.694±.003	0.817±.003	0.877±.045	0.762±.007
	𝒢-VAE _+exp_	0.642±.003	0.784±.002	0.863±.032	0.737±.008
