# Supplementary material for: Poisson Variational Autoencoder
Source: ArXiv. 2024 Dec 9:arXiv:2405.14473v2. Preprint. [Version 2] (PMC11661288)
Supplement: Supplement 1 [file NIHPP2405.14473v2-supplement-1.pdf]

A Are real neurons truly Poisson?

In this section, we discuss empirical and theoretical observations from neuroscience that motivated our Poisson assumption.

"Poisson-like" noise in neuroscience has a long history. It begins with observations that neurons do not fire the same sequence of spikes to repeated presentations of the same input, and that the variance is proportional to the mean [128, 129], and was followed by the observation that for short counting windows, that proportionality is one [49, 50, 130–132]. Larger windows and higher visual areas are notably super-Poisson, but that can be attributed to a modulation of the rate of an inhomogeneous Poisson process [51].

In other words, neurons are conditionally Poisson, not marginally Poisson [133].

Spike-generation, it is argued, is not noisy [134–136], but synaptic noise [137], or noise on the membrane potential, can create a Poisson-like distribution of spikes [138]. An important caveat is that the well-known example of precision in spike generation by Mainen and Sejnowski [134] is effectively captured by a Poisson-process Generalized Linear Model (GLM; Weber and Pillow [139]). However, this precision relies on a Bernoulli approximation to a Poisson process, allowing only 0 or 1 spikes. There is a widely-held misconception that precise timing cannot be produced by spike-rate models, but inhomogeneous rate models can produce precise spiking patterns at high time resolution [140]. In contrast, recent work has shown that correlations in excitatory inputs drive Poisson-like variability, explaining the widespread observation of Poisson-like noise in real neurons [141].

In summary, neurons are not literally Poisson, but it is a good choice. To set up the ELBO, one has to choose an approximate posterior and prior. Because spike counts are integer and cannot be negative, Poisson is a more natural choice than Gaussian without knowing anything about neural firing statistics. Here, we found that the Poisson assumption led to a model with interesting theoretical and empirical properties, where sparse coding emerged from the ELBO with Poisson.

Extending the $\mathcal{P}$-VAE to hierarchical architectures [2, 33, 34, 142] will make the latents conditionally Poisson, but not marginally Poisson (as they are modulated by top-down rates). Further extensions could implement doubly-stochastic spike generation [51, 143].

B Full derivations

In this section, we provide a self-contained and pedagogical introduction to VAEs, derive the $\mathcal{P}$-VAE loss function, and highlight how combining Poisson-distributed latents with predictive coding leads to the emergence of a metabolic cost term in the $\mathcal{P}$-VAE loss. For the case of a linear decoder, the reconstruction loss assumes a closed-form solution. This means we can compute the gradients analytically, which we can then use to evaluate the Poisson reparameterization trick.

B.1 Deriving the evidence lower bound (ELBO) loss

For completeness, let's first go over the basics. This section will provide a quick refresher on variational inference and how to derive the VAE loss from scratch. Assume the data $\boldsymbol{x} \in \mathbb{R}^M$ and K-dimensional latent variables $\boldsymbol{z}$ are jointly distributed as $p(\boldsymbol{x}, \boldsymbol{z})$, with the data generated through the following process:

$$p(\boldsymbol{x}) = \int p(\boldsymbol{x}, \boldsymbol{z})\, d\boldsymbol{z} = \int p(\boldsymbol{x}|\boldsymbol{z}) p(\boldsymbol{z})\, d\boldsymbol{z}, \tag{6}$$

In Bayesian posterior inference, the goal is to identify which latents $\boldsymbol{z}$ are likely given data $\boldsymbol{x}$. In other words, we want to approximate $P(\boldsymbol{z}|\boldsymbol{x})$, the optimal but (typically) intractable posterior distribution.

B.1.1 Variational inference and VAE loss function

To achieve approximate Bayesian inference, a common approach is to define a family of variational densities $\mathcal{Q}$ and find a member $q(\boldsymbol{z}|\boldsymbol{x}) \in \mathcal{Q}$ such that it sufficiently approximates the optimal posterior [144]. We call $q(\boldsymbol{z}|\boldsymbol{x})$ the *approximate posterior*. The general aim of variational inference (`VI`) can be summarized as follows:

$$\texttt{VI}: \quad \text{find a } q(\boldsymbol{z}|\boldsymbol{x}) \in \mathcal{Q} \text{ such that } q(\boldsymbol{z}|\boldsymbol{x}) \text{ is a good approximation of } p(\boldsymbol{z}|\boldsymbol{x}). \tag{7}$$

The goodness of our approximate posterior, or its closeness to the true posterior, is measured using the Kullback-Leibler (KL) divergence:

$$q^* = \underset{q \in \mathcal{Q}}{\operatorname{argmin}} \, \mathcal{D}_{\mathrm{KL}}\Big(q(\boldsymbol{z}|\boldsymbol{x}) \,\|\, p(\boldsymbol{z}|\boldsymbol{x})\Big). \tag{8}$$

We cannot directly optimize eq. (8), because $p(\boldsymbol{z}|\boldsymbol{x})$ is often intractable. Instead, we rearrange some terms and arrive at the following loss function:

$$\mathcal{L}_{\mathrm{NELBO}}(q) = -\,\mathbb{E}_{\boldsymbol{z}\sim q(\boldsymbol{z}|\boldsymbol{x})}\Big[\log p(\boldsymbol{x}|\boldsymbol{z})\Big] + \mathcal{D}_{\mathrm{KL}}\Big(q(\boldsymbol{z}|\boldsymbol{x}) \,\|\, p(\boldsymbol{z})\Big). \tag{9}$$

NELBO stands for negative ELBO, also known as "variational free energy." Notably, finding a $q \in \mathcal{Q}$ that minimizes $\mathcal{L}_{\mathrm{NELBO}}(q)$ in eq. (9) is equivalent to finding the optimal q^* in eq. (8).

The first term in eq. (9), often called the reconstruction term, captures the likelihood of the observed data $\boldsymbol{x}$, given latents $\boldsymbol{z}$, under the approximate posterior. For all our VAE models, we assume a Gaussian conditional likelihood with a fixed variance, as is typically done in the literature. This approximates the reconstruction term as the mean squared error between input data and their reconstructed version. The second term, known as the KL term, is more interesting. This term can assume very different forms depending on the distribution used.

B.2 The KL term

In this section, we will derive closed-form expressions for the KL term for different choices of the distributions $q(\boldsymbol{z}|\boldsymbol{x})$ and $p(\boldsymbol{z})$. Specifically, we will focus on Gaussian and Poisson parameterizations.

Predictive coding assumption. We will draw inspiration from predictive coding and assume that the bottom-up inference pathway only encodes the residual information relative to the top-down, or predicted information. We will apply this idea to both Gaussian and Poisson cases, and find that only in the Poisson case, the outcome becomes interpretable and resembles sparse coding objective.

B.2.1 KL term derivation: Gaussian

Let $q(\boldsymbol{z}|\boldsymbol{x}) = \mathcal{N}(\boldsymbol{z}; \boldsymbol{\mu}_q(\boldsymbol{x}), \boldsymbol{\sigma}_q(\boldsymbol{x}))$ and $p(\boldsymbol{z}) = \mathcal{N}(\boldsymbol{z}; \boldsymbol{\mu}_p, \boldsymbol{\sigma}_p)$, where the mean and variance are either outputs of the encoder network or parameters of the decoder network.

Now, let us implement the predictive coding assumption, where the encoder only keeps track of residual information that is not already contained in the prior information. Mathematically, this idea can be formalized as follows:

$$\begin{aligned} \boldsymbol{\mu}_p &\to \boldsymbol{\mu}, \qquad & \boldsymbol{\mu}_q &\to \boldsymbol{\mu} + \boldsymbol{\delta\mu} \\ \boldsymbol{\sigma}_p &\to \boldsymbol{\sigma}, \qquad & \boldsymbol{\sigma}_q &\to \boldsymbol{\sigma} \cdot \boldsymbol{\delta\sigma} \end{aligned} \tag{10}$$

With these modifications, the Gaussians KL term becomes:

$$\mathcal{D}_{\mathrm{KL}}\left(q \,\|\, p\right) = \frac{1}{2}\left(\frac{\boldsymbol{\delta\mu}^2}{\boldsymbol{\sigma}^2} + \boldsymbol{\delta\sigma}^2 - \log \boldsymbol{\delta\sigma}^2 - \mathbf{1}\right). \tag{11}$$

In standard Gaussian VAEs, the prior has no learnable parameter. Instead, we have $\boldsymbol{\mu} \to \mathbf{0}$ and $\boldsymbol{\sigma} \to \mathbf{1}$. Therefore, the final form of the KL term for a standard Gaussian VAE is:

$$\mathcal{D}_{\mathrm{KL}}\left(q \,\|\, \mathcal{N}(\mathbf{0}, \mathbf{1})\right) = \frac{1}{2}\Big(\boldsymbol{\delta\mu}^2 + \boldsymbol{\delta\sigma}^2 - \log \boldsymbol{\delta\sigma}^2 - \mathbf{1}\Big). \tag{12}$$

We observe that the KL term vanishes when $\boldsymbol{\delta\mu} \to \mathbf{0}$ and $\boldsymbol{\delta\sigma} \to \mathbf{1}$. This happens whenever no new information is propagated through the encoder, a phenomenon known as posterior collapse.

Other than this trivial observation, eq. (12) does not really lend itself to interpretation. In contrast, will show below that a Poisson parameterization of VAEs leads to a much more interpretable outcome for the KL term.

B.2.2 KL term derivation: Poisson

Now suppose $q(z|\boldsymbol{x}) = \mathcal{P}\text{ois}(z; r\delta r(\boldsymbol{x}))$, and $p(z) = \mathcal{P}\text{ois}(z; r)$, where z is literally the spike count of a single latent dimension—or shall we say, neuron?

In the Poisson case, the KL term becomes more interpretable, as we will show below. Recall that the Poisson distribution for a single variable z, given rate $\lambda \in \mathbb{R}_{>0}$, is given by:

$$\mathcal{P}\text{ois}(z; \lambda) = \frac{\lambda^z e^{-\lambda}}{z!}. \tag{13}$$

Plug this expressions into the KL divergence definition to get:

$$\begin{aligned}
\mathcal{D}_{\text{KL}}(q \,\|\, p) &= \mathbb{E}_{z\sim q}\left[\log \frac{q}{p}\right] \\
&= \mathbb{E}_{z\sim q}\left[\log \frac{(r\delta r)^z e^{-r\delta r}/z!}{r^z e^{-r}/z!}\right] \\
&= \mathbb{E}_{z\sim q}\left[\log \left(\left(\frac{r\delta r}{r}\right)^z e^{-r\delta r + r}\right)\right] \\
&= \mathbb{E}_{z\sim q}\left[\log \delta r^z + \log e^{-r\delta r + r}\right] \\
&= \mathbb{E}_{z\sim q}\left[z \log \delta r - r\delta r + r\right] \\
&= \mathbb{E}_{z\sim q}\left[z\right] \log \delta r - r\delta r + r \\
&= r\delta r \log \delta r - r\delta r + r \\
&= r\left(1 - \delta r + \delta r \log \delta r\right) \\
&= r f(\delta r),
\end{aligned} \tag{14}$$

where we have define $f(y) := 1 - y + y \log y$.

To examine the behavior of the Poisson KL term, we assume $\delta r = 1 + \epsilon$, where $\epsilon \ll 1$, then Taylor expand f. Calculating the first and second derivatives of $f(y) = 1 - y + y \log y$ gives $f'(y) = \log y$ and $f''(y) = 1/y$. Thus:

$$\begin{aligned}
f(1+\epsilon) &= f(1) + \epsilon f'(1) + \frac{\epsilon^2}{2!} f''(1) + \mathcal{O}(\epsilon^3) \\
&= 0 + 0 + \frac{\epsilon^2}{2!} + \mathcal{O}(\epsilon^3) \\
&\approx \frac{1}{2}\epsilon^2
\end{aligned} \tag{15}$$

Plug this back into eq. (14) to get:

$$\begin{aligned}
\mathcal{D}_{\text{KL}}(q \,\|\, p) &= r f(\delta r) \\
&= r f(1+\epsilon) \\
&\approx \frac{1}{2} r \epsilon^2.
\end{aligned} \tag{16}$$

For small deviations ϵ, the KL term simplifies to the product of the prior firing rate, r, and ϵ^2. See Fig. 6 for a visualization of the full function, $f(\delta r) = 1 - \delta r + \delta r \log \delta r$, along with its quadratic approximation near $\delta r = 1$.

In general, there are two ways to minimize the KL term: dead prior neurons ($r \to 0$), or posterior collapse ($\delta r \to 1$).

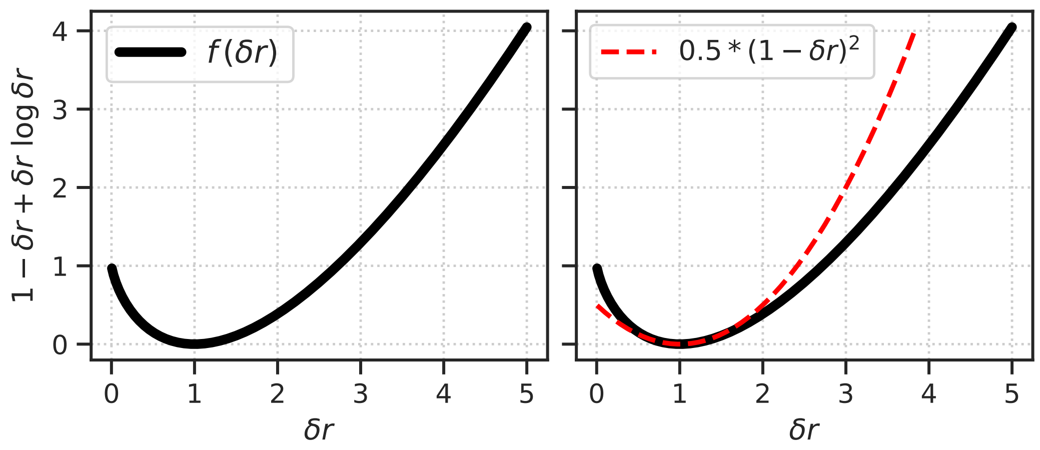

Figure 6: Left, residual term $f(\delta r)$ from eq. (14). Right, quadratic approximation of f from eq. (15).

Together with the reconstruction loss, the NELBO for a 1-dimensional $\mathcal{P}$-VAE reads:

$$\mathcal{L}_{\text{PVAE}}(r, \delta r) = \mathcal{L}_{\text{recon.}}(r, \delta r) + r\,(1 - \delta r + \delta r \log \delta r)\,. \tag{17}$$

Finally, it is easy to show that for K-dimensional latent space, eq. (14) generalizes to:

$$\mathcal{D}_{\text{KL}}\Big(\mathcal{P}\text{ois}(\boldsymbol{z}; \boldsymbol{r} \odot \boldsymbol{\delta r}(\boldsymbol{x})) \,\|\, \mathcal{P}\text{ois}(\boldsymbol{z}; \boldsymbol{r})\Big) = \boldsymbol{r} \cdot f(\boldsymbol{\delta r}), \tag{18}$$

where $\odot$ and $\cdot$ denote the Hadamard (element-wise) and vector products, respectively.

B.3 Connection to sparse coding

Equation (17) mirrors sparse coding due to the presence of the firing rate in the objective function. Furthermore, it follows the principle of predictive coding by design. Thus, our Poisson formulation of VAEs effectively unifies these two major themes in theoretical neuroscience. Let's explore this curious connection to sparse coding more closely below.

B.4 Statistically independent neurons

Suppose our $\mathcal{P}$-VAE has K statistically independent neurons, and $\boldsymbol{z} \in \mathbb{Z}_{\geq 0}^K$ is the spike count variable, where $\mathbb{Z}_{\geq 0} = \{0, 1, 2, \ldots\}$ is the set of non-negative integers. Let us use bold font $\boldsymbol{r}$ and $\boldsymbol{\delta r}$ to refer to the firing rate vectors of the representation and error units, respectively. Recall that we allowed these variables to interact in a multiplicative way to construct the posterior rates, $\lambda_i(\boldsymbol{x}) = r_i \delta r_i(\boldsymbol{x})$. More explicitly, we have:

$$\begin{aligned} q(\boldsymbol{z}|\boldsymbol{x}) &= \mathcal{P}\text{ois}(\boldsymbol{z}; \boldsymbol{r} \odot \boldsymbol{\delta r}) = \prod_{i=1}^{K} \mathcal{P}\text{ois}(z_i; r_i \delta r_i) = \prod_{i=1}^{K} \frac{\lambda_i^{z_i} e^{-\lambda_i}}{z_i!}, \\ p(\boldsymbol{z}) &= \mathcal{P}\text{ois}(\boldsymbol{z}; \boldsymbol{r}) = \prod_{i=1}^{K} \mathcal{P}\text{ois}(z_i; r_i) = \prod_{i=1}^{K} \frac{r_i^{z_i} e^{-r_i}}{z_i!}. \end{aligned} \tag{19}$$

Note that, unlike a standard Gaussian VAE, the prior in $\mathcal{P}$-VAE is parameterized using $\boldsymbol{r}$, which is learned from data along with the other parameters. Similar to standard Gaussian VAEs, $\boldsymbol{\delta r}(\boldsymbol{x})$ is parameterized as a neural network.

B.5 Linear decoder

Following the sparse coding literature [3], we will now assume our decoder generates the input image $\boldsymbol{x} \in \mathbb{R}^M$ as a linear sum of K basis elements, $\boldsymbol{\Phi} \in \mathbb{R}^{M \times K}$. Additionally, we choose a diagonal Gaussian distribution with fixed variance as our conditional likelihood, resulting in a mean squared error between the input $\boldsymbol{x}$, and its reconstruction $\boldsymbol{\Phi z}$.

Given these assumptions, the reconstruction loss for a VAE with approximate posterior q can be expressed as follows:

$$\mathcal{L}_{\text{recon.}}(\boldsymbol{x}; q) = \mathbb{E}_{\boldsymbol{z} \sim q(Z|X=\boldsymbol{x})}\left[\|\boldsymbol{x} - \boldsymbol{\Phi z}\|_2^2\right]. \tag{20}$$

For a linear decoder, the reconstruction term $\|\boldsymbol{x} - \boldsymbol{\Phi z}\|_2^2$ contains only the first and second moments of $\boldsymbol{z}$. Consequently, the expectation in eq. (20) can be analytically computed. This results in a close-form expression for the reconstruction loss, and consequently, its gradients as well.

In general, whenever the VAE decoder is linear, the following result holds:

$$\boxed{\mathcal{L}_{\text{recon.}}(\boldsymbol{x}; q, \boldsymbol{\Phi}) = \|\boldsymbol{x} - \boldsymbol{\Phi}\,\mathbb{E}_q[Z]\|_2^2 + \text{Var}_q[Z]^T \text{diag}(\boldsymbol{\Phi}^T\boldsymbol{\Phi}).} \tag{21}$$

Note that a linear decoder is the only assumption we needed to obtain this closed-form solution. There are no restrictions on the form of the encoder: it can be linear, or as complicated as we want. We only have to compute the mean and variance of the posterior.

Specifically, for the Poisson case, we only need to know the following expectation values:

$$\begin{aligned} \mathbb{E}_{\boldsymbol{z} \sim \mathcal{P}\text{ois}(\boldsymbol{z};\boldsymbol{\lambda})}\left[z_i\right] &= \lambda_i, \\ \mathbb{E}_{\boldsymbol{z} \sim \mathcal{P}\text{ois}(\boldsymbol{z};\boldsymbol{\lambda})}\left[z_i z_j\right] &= \lambda_i\lambda_j + \delta_{ij}\lambda_i. \end{aligned} \tag{22}$$

Here are the reconstruction losses for both Poisson and Gaussian VAEs with linear decoders, put side-by-side for comparison:

$$\boxed{\begin{array}{ll} \text{Poisson:} & \mathcal{L}_{\text{recon.}}(\boldsymbol{x}; \boldsymbol{\lambda}, \boldsymbol{\Phi}) = \|\boldsymbol{x} - \boldsymbol{\Phi\lambda}\|_2^2 + \boldsymbol{\lambda}^T \text{diag}(\boldsymbol{\Phi}^T\boldsymbol{\Phi}), \\ \text{Gaussian:} & \mathcal{L}_{\text{recon.}}(\boldsymbol{x}; \boldsymbol{\mu}, \boldsymbol{\sigma}, \boldsymbol{\Phi}) = \|\boldsymbol{x} - \boldsymbol{\Phi\mu}\|_2^2 + (\boldsymbol{\sigma}^2)^T \text{diag}(\boldsymbol{\Phi}^T\boldsymbol{\Phi}). \end{array}} \tag{23}$$

Given these assumptions, the NELBO (eq. (9)) for $\mathcal{P}$-VAE with a linear decoder becomes:

$$\boxed{\mathcal{L}_{\text{SC-PVAE}}(\boldsymbol{x}; \boldsymbol{\delta r}, \boldsymbol{r}, \boldsymbol{\Phi}) = \|\boldsymbol{x} - \boldsymbol{\Phi\lambda}\|_2^2 + \boldsymbol{\lambda}^T \text{diag}(\boldsymbol{\Phi}^T\boldsymbol{\Phi}) + \beta \sum_{i=1}^{K} r_i f(\delta r_i).} \tag{24}$$

Recall that we have $f(y) = 1 - y + y \log y$ (see Fig. 6). We introduced the β term here to control the trade-off between the reconstruction and the `KL` term [102]. Additionally, we dropped the explicit dependence of $\boldsymbol{\delta r}(\boldsymbol{x})$ on the input image $\boldsymbol{x}$ to enhance readability.

B.6 Linear encoder

We can further simplify the $\mathcal{P}$-VAE architecture by making the encoder also linear. Let $\boldsymbol{W} \in \mathbb{R}^{K \times M}$ denote the encoder's weight matrix, and assume an exponential link function mapping the input to residual firing rates, i.e., $\boldsymbol{\delta r} = \exp(\boldsymbol{W x})$.

Starting from eq. (24), substituting $\log \boldsymbol{\delta r} = \boldsymbol{W x}$, and rearranging terms yields the following loss function for the $\langle \texttt{lin}|\texttt{lin} \rangle$ $\mathcal{P}$-VAE:

$$\mathcal{L}_{\text{Lin-PVAE}} = \boldsymbol{\lambda}^T\boldsymbol{\Phi}^T\boldsymbol{\Phi\lambda} + \boldsymbol{\lambda}^T \text{diag}(\boldsymbol{\Phi}^T\boldsymbol{\Phi} - \beta\boldsymbol{I}) + \boldsymbol{\lambda}^T(\beta\boldsymbol{W} - 2\boldsymbol{\Phi}^T)\boldsymbol{x} + \beta\sum_{i=1}^{K} r_i + \boldsymbol{x}^T\boldsymbol{x}. \tag{25}$$

C Architecture, training, and hyperparameter details

C.1 Datasets: additional details

We consider three datasets in this paper. We tile up the van Hateren dataset of natural images [104] and CIFAR10 into 16×16 patches and apply whitening and contrast normalization using the code made available by Boutin et al. [105]. This operation results in the following total number of samples:

- **van Hateren**: $\#$train $= 107{,}520$, $\#$validation $= 28{,}224$,
- **CIFAR**$_{16\times16}$: $\#$train $= 200{,}000$, $\#$validation $= 40{,}000$.

We use the MNIST dataset primarily for the downstream classification task. After the training is done, we use the following train/validation split to evaluate the models:

- **K-nearest neighbor classification** (tables 4 and 6): For this task, we only make use of the validation set for both training and testing of the classifier. We divide up the $N = 10{,}000$ validation samples into two disjoint sets of $N = 5{,}000$ samples each. We then draw random samples (without replacement) from the first half and use them for training the KNN classifier. We then test the performance on the other half.
- **Shattering dimensionality** (tables 4 and 6, last column): We use the entire MNIST training set ($N = 60{,}000$ samples) to train logistic regression classifiers on extracted representations. We then test the results using the entire validation set ($N = 10{,}000$ samples).

C.2 Architecture details

For sparse coding results, we focused on models with linear decoders. For the fully linear models (Figs. 4 and 10) both the encoder and decoder were linear layers, without bias.

For the convolutional components, we use residual layers without batch norm. For van Hateren and CIFAR$_{16\times16}$ datasets, the encoders had 5 layers ($2 \times \mathrm{conv}$ each). The decoders had 8 convolutional layers ($1 \times \mathrm{conv}$ each). For the MNIST dataset, the encoders had 7 layers ($2 \times \mathrm{conv}$ each). The decoders had 10 convolutional layers ($1 \times \mathrm{conv}$ each). For all convolutional encoders, the output from ResNet was followed by a learned pooling layer. The pooled output was then fed into a feed-forward layer inspired by Transformers [145], which includes a layer norm as the final operation, the output of which was fed into a linear layer that projects features into posterior distribution parameters. For all convolutional decoders, nearest neighbor upsampling was performed to scale up the spatial dimension of reconstructions, as suggested by Child [34].

We experimented with both leaky_relu and swish activation functions [146, 147], and found that swish consistently outperformed leaky_relu in all our experiments across datasets and VAE models.

Please see our code for the full architecture details.

C.3 Training details

We used a variety of learning rates and batch sizes, depending on the dataset and architecture. For $\langle \texttt{lin}|\texttt{lin} \rangle$ and $\langle \texttt{conv}|\texttt{lin} \rangle$ models, we used $lr = 0.005$, and for $\langle \texttt{conv}|\texttt{conv} \rangle$ models we used $lr = 0.002$. All models were trained using the AdaMax optimizer [148] with a cosine learning rate schedule [149]. Please see our code for the full details of training hyperparameters. Overall, we trained 195 VAE models, $n = 5$ seeds each, resulting in a total of $195 \times 5 = 975$ VAEs. For sparse coding models, we ran ISTA [81, 82] and LCA [80] with 270 hyperparameter combinations each. Training all models took roughly a week on 8 RTX 6000 Ada GPUs.

Temperature annealing for discrete VAEs. We also annealed the temperature from a large value to a smaller value during the same first half of training for $\mathcal{P}$-VAE and $\mathcal{C}$-VAE. We found that the specific functional form of temperature annealing (e.g., linear, exponential, etc.) did not matter as much as the final temperature (Fig. 9). For both $\mathcal{P}$-VAE and $\mathcal{C}$-VAE, we start from $T_{\text{start}} = 1.0$ and anneal down to $T_{\text{stop}} = 0.05$ for $\mathcal{P}$-VAE, and $T_{\text{stop}} = 0.1$ for $\mathcal{C}$-VAE. We found that the $\mathcal{C}$-VAE performance was not very sensitive to the choice of T_{stop}, corroborating previous reports [89, 98].

The $\mathcal{P}$-VAE was relatively more sensitive to the value of T_{stop}, and we found marginal improvements when reducing it from 0.1 to 0.05. See Fig. 9 for comprehensive experiments exploring the effect of the final temperature, as well as a "hard-forward" training method where we set $T = 0$ in the forward pass (ensuring integer samples) and use a non-zero T only during the backward pass (surrogate gradients). We find that our "relaxed Poisson" approach (Fig. 3) consistently outperforms the hard-forward approach.

KL annealing for VAEs. For all VAE models, we annealed the `KL` term during the first half of the training, which is known to be an effective trick in training VAEs [2, 33, 142, 150, 151].

C.3.1 Training: sparse coding models

To fit LCA and ISTA models, we explored a combination of 6 β schedules (same β as in eq. (1)), 3 numbers of iteration (for inference), 3 learning rates, and 5 different seeds (for dictionary initialization). The code for LCA was obtained from the public python library "lca-pytorch" ([152]), and the code for ISTA was obtained from public "sparsecoding" repository of the Redwood Center for Theoretical Neuroscience (with added clipping of coefficients to be nonnegative, following the thresholding step).

We explored learning rates of 1×10^{-1}, 1×10^{-2}, and 1×10^{-3}. We trained all models for 100 epochs. We scheduled the β parameters linearly, starting from β_{start}, and stepped it up every five epochs by β_{step}, until it reached β_{end}. We explored the following β schedules (expressed as β_{start}:β_{end}:β_{step}):

$$0.05{:}0.7{:}0.1, \quad 0.01{:}0.1{:}0.01, \quad 0.1{:}1.0{:}0.1, \quad 0.05{:}0.7{:}0.05, \quad 0.05{:}0.5{:}0.05, \quad 0.1{:}0.1{:}0$$

We also explored the inference iteration limits of 100, 500, and 900 iterations. We selected the best fits to include in the main results shown in Figs. 4 and 5.

D Supplementary results

In this section, we include additional results that further support those reported in the main paper, including:

Table 5 contains the negative ELBO values for all VAE models with a linear decoder. This table reveals a comparable performance between using Monte Carlo samples to estimate gradients, versus optimizing the exact loss (see eqs. (4), (21), (23) and (24)), highlighting the effectiveness of our Poisson reparameterization algorithm.

Figure 7 uses the same data from the main paper Table 2 to visualize the effects.

Figure 8 shows the dependence of loss on latent dimensionality. We find that increasing the number of latent dimensions consistently improves ELBO for ⟨`conv`|`lin`⟩ architectures, but ⟨`lin`|`lin`⟩ models either overfit (for van Hateren) or fail to improve (for CIFAR$_{16\times16}$) once K becomes large.

Figure 9 demonstrates the robustness of our Poisson reparameterization trick (Algorithm 1) to variations in the temperature parameter. Importantly, we also explore a "hard-forward" training approach, where we fix $T = 0$ during the forward pass but allow $T > 0$ in the backward pass. This is also known as *surrogate gradients*. We find that, somewhat surprisingly, this hard-forward method performs significantly worse than our "relaxed Poisson" approach (Fig. 3).

Figure 10 shows how the distribution of `KL` values (or the norm of decoder weights in the case of linear decoders) can be used to determine dead neurons that don't contribute to the encoding of information.

Table 6 contains the full set of downstream classification results. Related to Table 4.

Figure 11 shows the performance of a simple linear classifier (logistic regression) trained on unsupervised representations learned by various ⟨`conv`|`conv`⟩ VAEs. We find that increasing the latent dimension (K) generally improves the performance of $\mathcal{P}$-VAE, but at lower dimensions, other methods like $\mathcal{L}$-VAE and $\mathcal{G}$-VAE can outperform it.

Figure 12 shows MNIST samples generated from the latent space of different ⟨`conv`|`conv`⟩ VAE models, as well as their reconstruction performance.

Table 5: The reparameterized gradient estimators work as well as exact ones, across datasets and encoder architectures (linear vs. conv). Note that exact gradients are only computable for linear decoders (see eqs. (21), (23) and (24)). The values are negative ELBO (lower is better), shown as mean±99% confidence interval calculated from $n = 5$ different random initializations. For MNIST, our use of Gaussian conditional likelihoods means the numerical performance values are not directly comparable to studies that use binarized MNIST with a cross-entropy decoder. EX, exact, MC, Monte-Carlo, ST, straight-through [107]. See also Table 2 and supplementary Fig. 7.

Model		van Hateren		CIFAR$_{16\times16}$		MNIST	
		⟨lin|lin⟩	⟨conv|lin⟩	⟨lin|lin⟩	⟨conv|lin⟩	⟨lin|lin⟩	⟨conv|lin⟩
$\mathcal{P}$-VAE	EX	168.0±.8	162.4±.2	167.1±.2	162.1±.1	41.5±.1	39.7±.2
	MC	167.2±.1	163.4±.1	167.3±.1	162.9±.2	41.7±.2	40.1±.2
	ST	179.3±.1	179.4±.1	182.3±.1	182.3±.2	44.8±.1	44.2±.1
$\mathcal{G}$-VAE	EX	160.3±.1	154.4±.1	165.9±.1	149.2±.0	40.6±.1	40.0±.1
	MC	160.3±.1	154.4±.1	165.9±.1	149.2±.1	40.7±.1	40.1±.0
$\mathcal{C}$-VAE	EX	174.9±.1	186.3±.8	177.1±.1	180.6±.5	56.1±.7	59.1±.0
	MC	170.5±.1	171.9±.2	174.7±.1	176.5±.1	39.7±.2	59.1±.0
	ST	174.2±.2	181.1±.3	180.2±.0	185.6±.2	49.3±.1	63.8±3.4
$\mathcal{L}$-VAE	EX	167.3±.0	159.0±.2	170.1±.1	154.3±.1	42.1±.1	41.0±.0
	MC	167.3±.0	159.2±.2	170.1±.1	154.5±.1	42.1±.0	41.0±.0

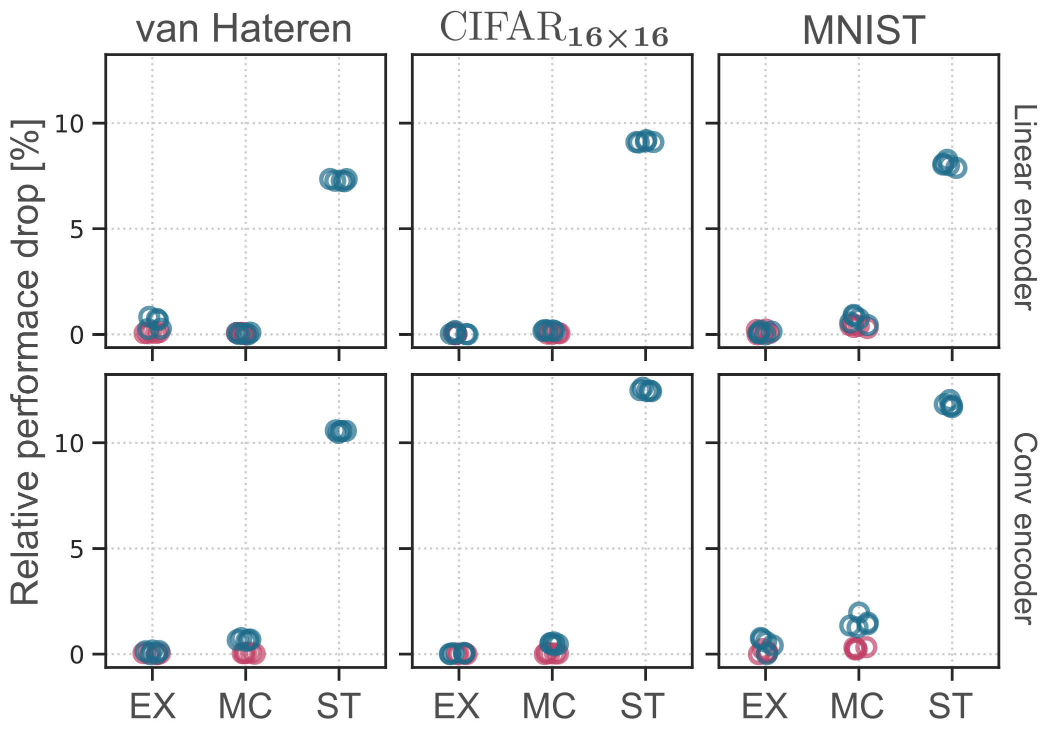

Figure 7: Performance drop relative to the best fit. Blue circles indicate $\mathcal{P}$-VAE results, red circles indicate $\mathcal{G}$-VAE results, and each set of $n = 5$ circles corresponds to five random initializations. Using Monte Carlo samples [153] and our Poisson reparameterization trick (Algorithm 1) to estimate gradients performs comparably to using exact gradients (see eqs. (21), (23) and (24)). Table 2 provides a tabular summary of these results. EX, exact, MC, Monte-Carlo, ST, straight-through [107].

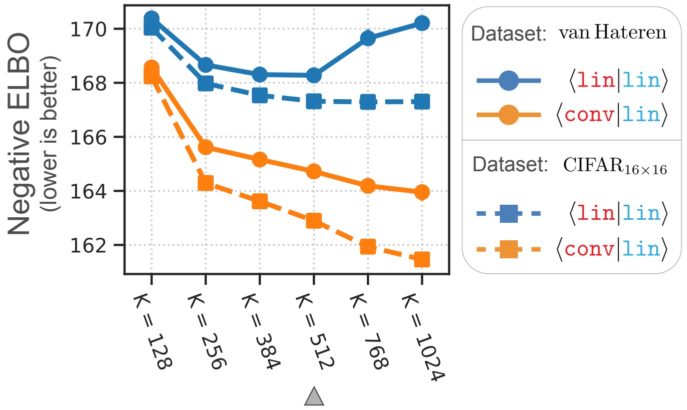

Figure 8: The effect of latent dimensionality on model performance across datasets and encoder architectures. For all convolutional encoder cases, ELBO improves as a function of latent dimensionality. However, for linear encoders, we see that the van Hateren dataset starts to overfit for $K > 512$, and it stagnates for the CIFAR$_{16\times16}$ dataset. In conclusion, more expressive encoders can find nonlinear features, represented using additional latent dimensions, but simple linear encoders struggle to utilize additional dimensions. The gray triangle indicates the setting used in the main results.

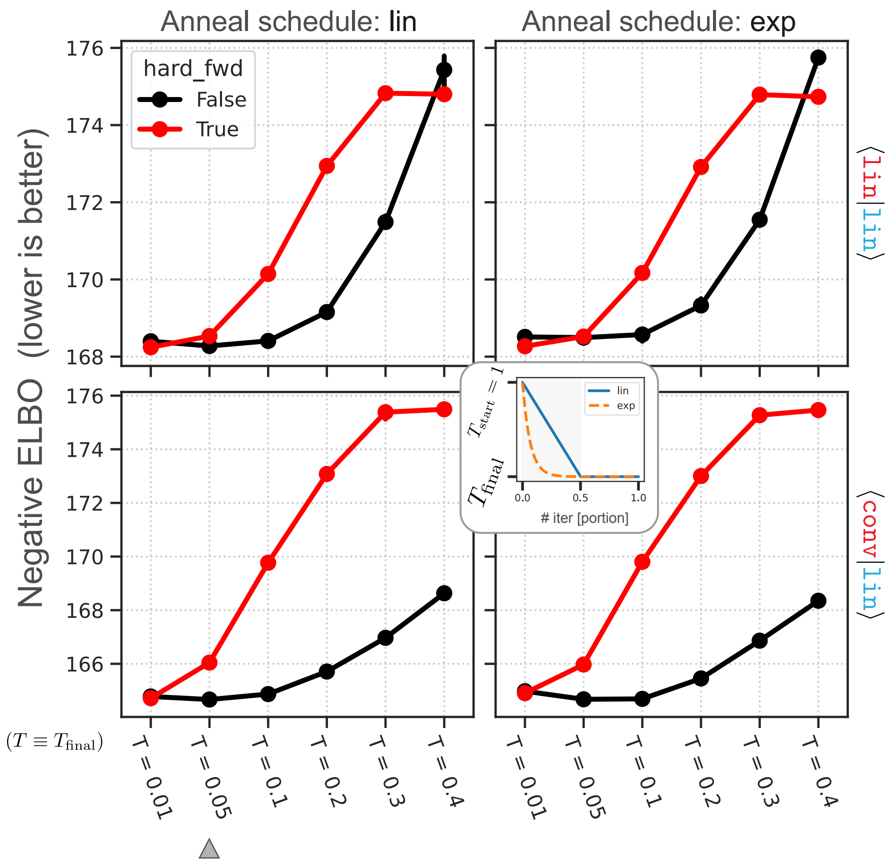

Figure 9: Performance as a function of the final temperature (T_{final}), annealing schedule (linear vs. exponential; inset), and the "hard-forward" approach. The hard-forward approach uses exact integer samples ($T = 0$) in the forward pass and applies nonzero temperatures only in the backward pass (i.e., "surrogate gradients"). Although all results are evaluated at $T = 0$ during testing, the hard-forward approach still underperforms our "relaxed Poisson" method (Fig. 3), which employs continuous (floating) samples during training due to a non-zero T (Algorithm 1). The gray triangle indicates the setting used in the main results: $T_{\text{final}} = 0.05$ with a linear annealing schedule.

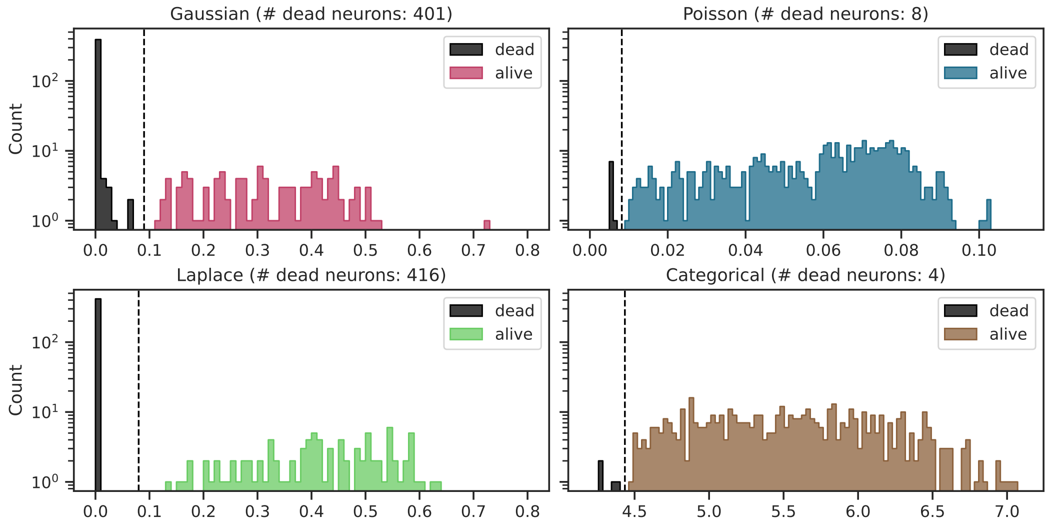

Figure 10: Identifying dead neurons using a histogram-based method. We bin the KL values and determine the gap between small values and larger ones. We identify neurons with KL values lower than the identified threshold (black dashed lines) and pronounce them dead. The figure shows the distribution of KL values over all neurons ($K = 512$) for $\mathcal{P}$-VAE, $\mathcal{G}$-VAE, and $\mathcal{L}$-VAE. The KL term is a single number for the $\mathcal{C}$-VAE because its latent space consists of a single one-hot categorical distribution with $K = 512$ categories. Therefore, for the $\mathcal{C}$-VAE, we use the distribution of decoder weight norms instead. These are the same models shown in Fig. 4, where both encoder and decoder are linear. Table 3 uses this method to quantify the proportion of active neurons for VAEs across different datasets and the choice of encoder architectures.

Table 6: Geometry of representations. Full set of results. Related to Table 4.

Latent dim.	Model	KNN classification (N, # labeled samples)			Shattering dim.
		$N = 200$	$N = 1{,}000$	$N = 5{,}000$	
$K = 10$	$\mathcal{P}$-VAE	$\mathbf{0.815}_{\pm.002}$	$\mathbf{0.919}_{\pm.001}$	$\mathbf{0.946}_{\pm.017}$	$\mathbf{0.797}_{\pm.009}$
	$\mathcal{C}$-VAE	$0.705_{\pm.002}$	$0.800_{\pm.002}$	$0.853_{\pm.040}$	$\mathbf{0.795}_{\pm.006}$
	$\mathcal{L}$-VAE	$0.757_{\pm.003}$	$0.869_{\pm.002}$	$\mathbf{0.924}_{\pm.028}$	$0.751_{\pm.008}$
	$\mathcal{G}$-VAE	$0.673_{\pm.003}$	$0.813_{\pm.002}$	$0.891_{\pm.033}$	$0.758_{\pm.007}$
	$\mathcal{G}$-VAE $_{+\text{relu}}$	$0.694_{\pm.003}$	$0.817_{\pm.003}$	$0.877_{\pm.045}$	$0.762_{\pm.007}$
	$\mathcal{G}$-VAE $_{+\text{exp}}$	$0.642_{\pm.003}$	$0.784_{\pm.002}$	$0.863_{\pm.032}$	$0.737_{\pm.008}$
$K = 50$	$\mathcal{P}$-VAE	$\mathbf{0.825}_{\pm.002}$	$\mathbf{0.927}_{\pm.001}$	$\mathbf{0.957}_{\pm.005}$	$\mathbf{0.935}_{\pm.003}$
	$\mathcal{C}$-VAE	$0.770_{\pm.002}$	$0.880_{\pm.001}$	$0.920_{\pm.009}$	$0.899_{\pm.004}$
	$\mathcal{L}$-VAE	$0.710_{\pm.003}$	$0.836_{\pm.003}$	$0.902_{\pm.038}$	$0.770_{\pm.007}$
	$\mathcal{G}$-VAE	$0.604_{\pm.003}$	$0.746_{\pm.002}$	$0.837_{\pm.022}$	$0.743_{\pm.007}$
	$\mathcal{G}$-VAE $_{+\text{relu}}$	$0.710_{\pm.002}$	$0.844_{\pm.002}$	$0.904_{\pm.026}$	$0.786_{\pm.006}$
	$\mathcal{G}$-VAE $_{+\text{exp}}$	$0.694_{\pm.003}$	$0.836_{\pm.002}$	$0.906_{\pm.027}$	$0.762_{\pm.007}$
$K = 100$	$\mathcal{P}$-VAE	$\mathbf{0.807}_{\pm.002}$	$\mathbf{0.925}_{\pm.001}$	$\mathbf{0.958}_{\pm.013}$	$\mathbf{0.949}_{\pm.002}$
	$\mathcal{C}$-VAE	$0.753_{\pm.002}$	$0.876_{\pm.001}$	$0.925_{\pm.005}$	$0.884_{\pm.004}$
	$\mathcal{L}$-VAE	$0.701_{\pm.004}$	$0.830_{\pm.003}$	$\mathbf{0.896}_{\pm.046}$	$0.767_{\pm.007}$
	$\mathcal{G}$-VAE	$0.636_{\pm.003}$	$0.789_{\pm.002}$	$0.875_{\pm.024}$	$0.763_{\pm.007}$
	$\mathcal{G}$-VAE $_{+\text{relu}}$	$0.757_{\pm.002}$	$0.881_{\pm.001}$	$\mathbf{0.933}_{\pm.019}$	$0.818_{\pm.006}$
	$\mathcal{G}$-VAE $_{+\text{exp}}$	$0.695_{\pm.003}$	$0.846_{\pm.002}$	$0.918_{\pm.024}$	$0.793_{\pm.006}$

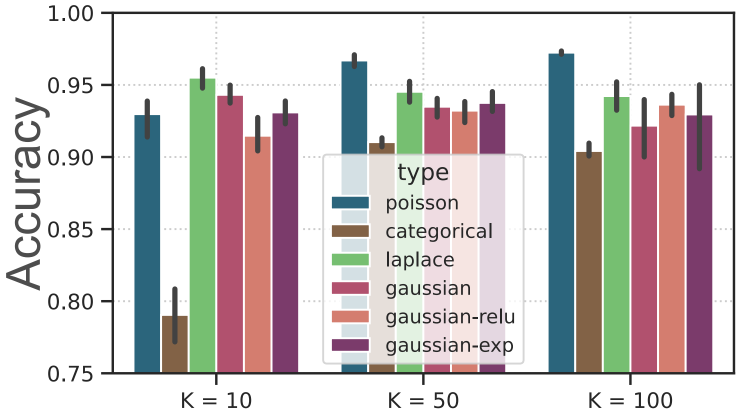

Figure 11: Downstream classification performance using a simple linear classifier. After unsupervised training of ⟨conv|conv⟩ VAEs on MNIST, we extracted latent representations and applied logistic regression. For $K = 100$, $\mathcal{P}$-VAE achieves the highest accuracy, while for $K = 10$, both $\mathcal{L}$-VAE and $\mathcal{G}$-VAE outperform it.

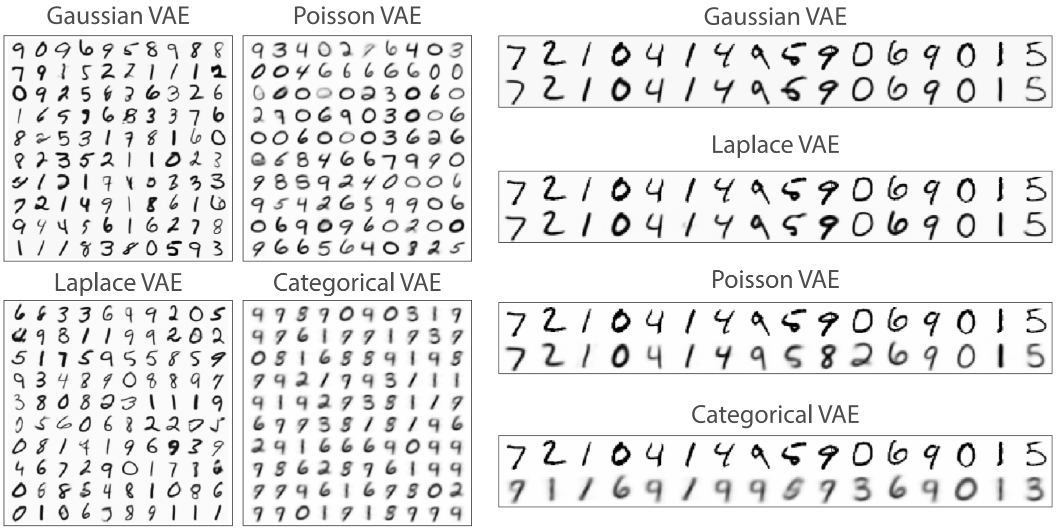

Figure 12: Generated samples (left) and reconstruction performance (right). These results shown here are from models with a ⟨conv|conv⟩ architectures and latent dimensionality of $K = 10$.

NeurIPS Paper Checklist

1. **Claims**

   Question: Do the main claims made in the abstract and introduction accurately reflect the paper's contributions and scope?

   Answer: [Yes]

   Justification: we provide comprehensive theoretical and empirical evidence to support our claims of (1) introducing the $\mathcal{P}$-VAE and its reparameterization trick; (2) $\mathcal{P}$-VAE containing amortized sparse coding as a special case; (3) $\mathcal{P}$-VAE largely avoiding posterior collapse; and (4) $\mathcal{P}$-VAE facilitating linear separability of categories at better sample efficiency, in sections 3 and 4, and supplemental appendices B to D.

   Guidelines:

   - The answer NA means that the abstract and introduction do not include the claims made in the paper.
   - The abstract and/or introduction should clearly state the claims made, including the contributions made in the paper and important assumptions and limitations. A No or NA answer to this question will not be perceived well by the reviewers.
   - The claims made should match theoretical and experimental results, and reflect how much the results can be expected to generalize to other settings.
   - It is fine to include aspirational goals as motivation as long as it is clear that these goals are not attained by the paper.

2. **Limitations**

   Question: Does the paper discuss the limitations of the work performed by the authors?

   Answer: [Yes]

   Justification: The limitations of (1) Poisson possibly not being a perfect description of cortical activity, and (2) amortization gap, are shown explicitly and thoroughly discussed in sections 4 and 5. Specifically, we have a dedicated paragraph for limitations in section 5. We evaluated our claims using multiple well-known datasets such as the van Hateren natural images [104], CIFAR10, and MNIST, on tasks such as reconstruction, sparse coding, and classification.

   Guidelines:

   - The answer NA means that the paper has no limitation while the answer No means that the paper has limitations, but those are not discussed in the paper.
   - The authors are encouraged to create a separate "Limitations" section in their paper.
   - The paper should point out any strong assumptions and how robust the results are to violations of these assumptions (e.g., independence assumptions, noiseless settings, model well-specification, asymptotic approximations only holding locally). The authors should reflect on how these assumptions might be violated in practice and what the implications would be.
   - The authors should reflect on the scope of the claims made, e.g., if the approach was only tested on a few datasets or with a few runs. In general, empirical results often depend on implicit assumptions, which should be articulated.
   - The authors should reflect on the factors that influence the performance of the approach. For example, a facial recognition algorithm may perform poorly when image resolution is low or images are taken in low lighting. Or a speech-to-text system might not be used reliably to provide closed captions for online lectures because it fails to handle technical jargon.
   - The authors should discuss the computational efficiency of the proposed algorithms and how they scale with dataset size.
   - If applicable, the authors should discuss possible limitations of their approach to address problems of privacy and fairness.
   - While the authors might fear that complete honesty about limitations might be used by reviewers as grounds for rejection, a worse outcome might be that reviewers discover limitations that aren't acknowledged in the paper. The authors should use their best

judgment and recognize that individual actions in favor of transparency play an important role in developing norms that preserve the integrity of the community. Reviewers will be specifically instructed to not penalize honesty concerning limitations.

3. **Theory Assumptions and Proofs**

   Question: For each theoretical result, does the paper provide the full set of assumptions and a complete (and correct) proof?

   Answer: [Yes]

   Justification: We provide the full derivation of the $\mathcal{P}$-VAE loss function, which is self-contained in the paper (section 3) and supplement (appendix B).

   Guidelines:

   - The answer NA means that the paper does not include theoretical results.
   - All the theorems, formulas, and proofs in the paper should be numbered and cross-referenced.
   - All assumptions should be clearly stated or referenced in the statement of any theorems.
   - The proofs can either appear in the main paper or the supplemental material, but if they appear in the supplemental material, the authors are encouraged to provide a short proof sketch to provide intuition.
   - Inversely, any informal proof provided in the core of the paper should be complemented by formal proofs provided in appendix or supplemental material.
   - Theorems and Lemmas that the proof relies upon should be properly referenced.

4. **Experimental Result Reproducibility**

   Question: Does the paper fully disclose all the information needed to reproduce the main experimental results of the paper to the extent that it affects the main claims and/or conclusions of the paper (regardless of whether the code and data are provided or not)?

   Answer: [Yes]

   Justification: We disclose all details relating to the algorithm, including the optimization objective (eq. (3)), architecture and training details (appendix C), and pseudo-code for Poisson reparameterized sampling (Algorithm 1). In addition, we intend to release all code and data needed for replicating our work.

   Guidelines:

   - The answer NA means that the paper does not include experiments.
   - If the paper includes experiments, a No answer to this question will not be perceived well by the reviewers: Making the paper reproducible is important, regardless of whether the code and data are provided or not.
   - If the contribution is a dataset and/or model, the authors should describe the steps taken to make their results reproducible or verifiable.
   - Depending on the contribution, reproducibility can be accomplished in various ways. For example, if the contribution is a novel architecture, describing the architecture fully might suffice, or if the contribution is a specific model and empirical evaluation, it may be necessary to either make it possible for others to replicate the model with the same dataset, or provide access to the model. In general. releasing code and data is often one good way to accomplish this, but reproducibility can also be provided via detailed instructions for how to replicate the results, access to a hosted model (e.g., in the case of a large language model), releasing of a model checkpoint, or other means that are appropriate to the research performed.
   - While NeurIPS does not require releasing code, the conference does require all submissions to provide some reasonable avenue for reproducibility, which may depend on the nature of the contribution. For example
     (a) If the contribution is primarily a new algorithm, the paper should make it clear how to reproduce that algorithm.
     (b) If the contribution is primarily a new model architecture, the paper should describe the architecture clearly and fully.

(c) If the contribution is a new model (e.g., a large language model), then there should either be a way to access this model for reproducing the results or a way to reproduce the model (e.g., with an open-source dataset or instructions for how to construct the dataset).

(d) We recognize that reproducibility may be tricky in some cases, in which case authors are welcome to describe the particular way they provide for reproducibility. In the case of closed-source models, it may be that access to the model is limited in some way (e.g., to registered users), but it should be possible for other researchers to have some path to reproducing or verifying the results.

5. **Open access to data and code**

   Question: Does the paper provide open access to the data and code, with sufficient instructions to faithfully reproduce the main experimental results, as described in supplemental material?

   Answer: [Yes]

   Justification: Our code, data, and model checkpoints are available from the following GitHub repository: https://github.com/hadivafaii/PoissonVAE.

   Guidelines:

   - The answer NA means that paper does not include experiments requiring code.
   - Please see the NeurIPS code and data submission guidelines (`https://nips.cc/public/guides/CodeSubmissionPolicy`) for more details.
   - While we encourage the release of code and data, we understand that this might not be possible, so "No" is an acceptable answer. Papers cannot be rejected simply for not including code, unless this is central to the contribution (e.g., for a new open-source benchmark).
   - The instructions should contain the exact command and environment needed to run to reproduce the results. See the NeurIPS code and data submission guidelines (`https://nips.cc/public/guides/CodeSubmissionPolicy`) for more details.
   - The authors should provide instructions on data access and preparation, including how to access the raw data, preprocessed data, intermediate data, and generated data, etc.
   - The authors should provide scripts to reproduce all experimental results for the new proposed method and baselines. If only a subset of experiments are reproducible, they should state which ones are omitted from the script and why.
   - At submission time, to preserve anonymity, the authors should release anonymized versions (if applicable).
   - Providing as much information as possible in supplemental material (appended to the paper) is recommended, but including URLs to data and code is permitted.

6. **Experimental Setting/Details**

   Question: Does the paper specify all the training and test details (e.g., data splits, hyperparameters, how they were chosen, type of optimizer, etc.) necessary to understand the results?

   Answer: [Yes]

   Justification: All details about how the data was used for training and testing, as well as which hyperparameters were used, are available at appendix C. In addition, the provided code replicates our results and therefore contains all details of implementation.

   Guidelines:

   - The answer NA means that the paper does not include experiments.
   - The experimental setting should be presented in the core of the paper to a level of detail that is necessary to appreciate the results and make sense of them.
   - The full details can be provided either with the code, in appendix, or as supplemental material.

7. **Experiment Statistical Significance**

   Question: Does the paper report error bars suitably and correctly defined or other appropriate information about the statistical significance of the experiments?

Answer: [Yes]

Justification: As stated in section 4, the paper reports confidence intervals and t-test significance tests, using false discovery rate (FDR) correction for multiple comparisons. The exact implementation details are included in the provided code for reproducibility.

Guidelines:

- The answer NA means that the paper does not include experiments.
- The authors should answer "Yes" if the results are accompanied by error bars, confidence intervals, or statistical significance tests, at least for the experiments that support the main claims of the paper.
- The factors of variability that the error bars are capturing should be clearly stated (for example, train/test split, initialization, random drawing of some parameter, or overall run with given experimental conditions).
- The method for calculating the error bars should be explained (closed form formula, call to a library function, bootstrap, etc.)
- The assumptions made should be given (e.g., Normally distributed errors).
- It should be clear whether the error bar is the standard deviation or the standard error of the mean.
- It is OK to report 1-sigma error bars, but one should state it. The authors should preferably report a 2-sigma error bar than state that they have a 96% CI, if the hypothesis of Normality of errors is not verified.
- For asymmetric distributions, the authors should be careful not to show in tables or figures symmetric error bars that would yield results that are out of range (e.g. negative error rates).
- If error bars are reported in tables or plots, The authors should explain in the text how they were calculated and reference the corresponding figures or tables in the text.

8. **Experiments Compute Resources**

   Question: For each experiment, does the paper provide sufficient information on the computer resources (type of compute workers, memory, time of execution) needed to reproduce the experiments?

   Answer: [Yes]

   Justification: We provide details about our compute resources (GPUs), and duration of training in section 4.

   Guidelines:

   - The answer NA means that the paper does not include experiments.
   - The paper should indicate the type of compute workers CPU or GPU, internal cluster, or cloud provider, including relevant memory and storage.
   - The paper should provide the amount of compute required for each of the individual experimental runs as well as estimate the total compute.
   - The paper should disclose whether the full research project required more compute than the experiments reported in the paper (e.g., preliminary or failed experiments that didn't make it into the paper).

9. **Code Of Ethics**

   Question: Does the research conducted in the paper conform, in every respect, with the NeurIPS Code of Ethics `https://neurips.cc/public/EthicsGuidelines`?

   Answer: [Yes]

   Justification: Our paper follows the code of ethics, including preserving anonymity (such as in releasing code anonymously).

   Guidelines:

   - The answer NA means that the authors have not reviewed the NeurIPS Code of Ethics.
   - If the authors answer No, they should explain the special circumstances that require a deviation from the Code of Ethics.

- The authors should make sure to preserve anonymity (e.g., if there is a special consideration due to laws or regulations in their jurisdiction).

10. **Broader Impacts**

    Question: Does the paper discuss both potential positive societal impacts and negative societal impacts of the work performed?

    Answer: [NA]

    Justification: Our paper is considered foundational research, and does not target practical tasks that can be deployed outside of the research field. Thus we do not anticipate negative social impacts from this work.

    Guidelines:

    - The answer NA means that there is no societal impact of the work performed.
    - If the authors answer NA or No, they should explain why their work has no societal impact or why the paper does not address societal impact.
    - Examples of negative societal impacts include potential malicious or unintended uses (e.g., disinformation, generating fake profiles, surveillance), fairness considerations (e.g., deployment of technologies that could make decisions that unfairly impact specific groups), privacy considerations, and security considerations.
    - The conference expects that many papers will be foundational research and not tied to particular applications, let alone deployments. However, if there is a direct path to any negative applications, the authors should point it out. For example, it is legitimate to point out that an improvement in the quality of generative models could be used to generate deepfakes for disinformation. On the other hand, it is not needed to point out that a generic algorithm for optimizing neural networks could enable people to train models that generate Deepfakes faster.
    - The authors should consider possible harms that could arise when the technology is being used as intended and functioning correctly, harms that could arise when the technology is being used as intended but gives incorrect results, and harms following from (intentional or unintentional) misuse of the technology.
    - If there are negative societal impacts, the authors could also discuss possible mitigation strategies (e.g., gated release of models, providing defenses in addition to attacks, mechanisms for monitoring misuse, mechanisms to monitor how a system learns from feedback over time, improving the efficiency and accessibility of ML).

11. **Safeguards**

    Question: Does the paper describe safeguards that have been put in place for responsible release of data or models that have a high risk for misuse (e.g., pretrained language models, image generators, or scraped datasets)?

    Answer: [NA]

    Justification: Our paper utilizes publicly domain datasets (not scraped), and poses no safety risks.

    Guidelines:

    - The answer NA means that the paper poses no such risks.
    - Released models that have a high risk for misuse or dual-use should be released with necessary safeguards to allow for controlled use of the model, for example by requiring that users adhere to usage guidelines or restrictions to access the model or implementing safety filters.
    - Datasets that have been scraped from the Internet could pose safety risks. The authors should describe how they avoided releasing unsafe images.
    - We recognize that providing effective safeguards is challenging, and many papers do not require this, but we encourage authors to take this into account and make a best faith effort.

12. **Licenses for existing assets**

    Question: Are the creators or original owners of assets (e.g., code, data, models), used in the paper, properly credited and are the license and terms of use explicitly mentioned and properly respected?

Answer: [Yes]

Justification: We properly cite papers that introduce algorithms (such as LCA), datasets (such as MNIST, CIFAR10, van Hateren), and code (such as LCA and ISTA).

Guidelines:

- The answer NA means that the paper does not use existing assets.
- The authors should cite the original paper that produced the code package or dataset.
- The authors should state which version of the asset is used and, if possible, include a URL.
- The name of the license (e.g., CC-BY 4.0) should be included for each asset.
- For scraped data from a particular source (e.g., website), the copyright and terms of service of that source should be provided.
- If assets are released, the license, copyright information, and terms of use in the package should be provided. For popular datasets, `paperswithcode.com/datasets` has curated licenses for some datasets. Their licensing guide can help determine the license of a dataset.
- For existing datasets that are re-packaged, both the original license and the license of the derived asset (if it has changed) should be provided.
- If this information is not available online, the authors are encouraged to reach out to the asset's creators.

13. **New Assets**

    Question: Are new assets introduced in the paper well documented and is the documentation provided alongside the assets?

    Answer: [Yes]

    Justification: New assets introduced in the paper consist of our codebase which includes notebooks to replicate our experiments and analyses, and contains documentation.

    Guidelines:

    - The answer NA means that the paper does not release new assets.
    - Researchers should communicate the details of the dataset/code/model as part of their submissions via structured templates. This includes details about training, license, limitations, etc.
    - The paper should discuss whether and how consent was obtained from people whose asset is used.
    - At submission time, remember to anonymize your assets (if applicable). You can either create an anonymized URL or include an anonymized zip file.

14. **Crowdsourcing and Research with Human Subjects**

    Question: For crowdsourcing experiments and research with human subjects, does the paper include the full text of instructions given to participants and screenshots, if applicable, as well as details about compensation (if any)?

    Answer: [NA]

    Justification: Our paper does not involve crowdsourcing nor research with human subjects.

    Guidelines:

    - The answer NA means that the paper does not involve crowdsourcing nor research with human subjects.
    - Including this information in the supplemental material is fine, but if the main contribution of the paper involves human subjects, then as much detail as possible should be included in the main paper.
    - According to the NeurIPS Code of Ethics, workers involved in data collection, curation, or other labor should be paid at least the minimum wage in the country of the data collector.

15. **Institutional Review Board (IRB) Approvals or Equivalent for Research with Human Subjects**

Question: Does the paper describe potential risks incurred by study participants, whether such risks were disclosed to the subjects, and whether Institutional Review Board (IRB) approvals (or an equivalent approval/review based on the requirements of your country or institution) were obtained?

Answer: [NA]

Justification: Our paper does not involve crowdsourcing nor research with human subjects.

Guidelines:

- The answer NA means that the paper does not involve crowdsourcing nor research with human subjects.
- Depending on the country in which research is conducted, IRB approval (or equivalent) may be required for any human subjects research. If you obtained IRB approval, you should clearly state this in the paper.
- We recognize that the procedures for this may vary significantly between institutions and locations, and we expect authors to adhere to the NeurIPS Code of Ethics and the guidelines for their institution.
- For initial submissions, do not include any information that would break anonymity (if applicable), such as the institution conducting the review.
